# Design, Synthesis and Biological Evaluation of novel Hedgehog Inhibitors for treating Pancreatic Cancer

**DOI:** 10.1038/s41598-017-01942-7

**Published:** 2017-05-10

**Authors:** Vinod Kumar, Amit Kumar Chaudhary, Yuxiang Dong, Haizhen A. Zhong, Goutam Mondal, Feng Lin, Virender Kumar, Ram I. Mahato

**Affiliations:** 1 Department of Pharmaceutical Sciences, University of Nebraska Medical Center, Omaha, NE 68198 USA; 2 Department of Chemistry, University of Nebraska at Omaha, 6001 Dodge Street, Omaha, NE 68182 USA

## Abstract

Hedgehog (Hh) pathway is involved in epithelial-mesenchymal transition (EMT) and cancer stem cell (CSC) maintenance resulting in tumor progression. GDC-0449, an inhibitor of Hh pathway component smoothened (Smo) has shown promise in the treatment of various cancers including pancreatic cancer. However, the emergence of resistance during GDC-0449 treatment with numerous side effects limits its use. Therefore, here we report the design, synthesis and evaluation of novel GDC-0449 analogs using *N*-[3-(2-pyridinyl) phenyl] benzamide scaffold. Cell-based screening followed by molecular simulation revealed 2-chloro-*N* ^1^-[4-chloro-3-(2-pyridinyl)phenyl]-*N* ^4^,*N* ^4^-bis(2-pyridinylmethyl)-1,4-benzenedicarboxamide (MDB5) as most potent analog, binding with an extra interactions in seven-transmembrane (7-TM) domain of Smo due to an additional 2-pyridylmethyl group than GDC-0449. Moreover, MDB5 was more efficient in inhibiting Hh pathway components as measured by Gli-1 and Shh at transcriptional and translational levels. Additionally, a significant reduction of ALDH1, CD44 and Oct-3/4, key markers of pancreatic CSC was observed when MIA PaCa-2 cells were treated with MDB5 compared to GDC-0449. In a pancreatic tumor mouse model, MDB5 containing nanoparticles treated group showed significant inhibition of tumor growth without loss in body weight. These evidence highlight the enhanced Hh pathway inhibition and anticancer properties of MDB5 leaving a platform for mono and/or combination therapy.

## Introduction

Hedgehog (Hh) signaling regulates cell growth, differentiation and plays a major role in homeostasis of various organs and tissues by affecting stem cells^1–3^. Aberrant activation of Hh pathway associates itself with a variety of human tumors resulting in tumorigenesis, malignancy and metastasis; which in normal cells is less active^2, 4, 5^. The first association of Hh signaling in cancer was established after Gorlin’s syndrome resulting from an autosomal loss of Patched (Ptch)^6^. Follow-up studies revealed activation of this pathway in basal cell carcinoma (BCC), medulloblastoma (MB), leukemia, breast, esophagus, gastric, pancreatic, prostate and small-cell lung cancers^7–13^. Hh signaling is a key regulator of physiological processes which plays a key role in cancer stem cell (CSC) biology^14^. Profound attention has been given to explore the role of CSC in the initiation and progression of solid malignancies. These cells are responsible for tumor onset, self-renewal, maintenance and metastasis due to their ability to express anti-apoptotic and drug resistant proteins, thus sustaining tumor growth^15, 16^. Conventional therapy merely kill most differentiated tumor cells except CSCs, which have intrinsic detoxifying mechanisms and can easily escape these therapies. CSCs and epithelial-mesenchymal transition (EMT) type cells have been proposed to play critical roles in chemoresistance and metastasis as demonstrated in several malignancies.

The development of small molecule targeting cancer-associated signaling pathways has been viewed as a promising approach for the treatment of solid tumors. Anticancer agents targeting several modules of the Hh pathway have been described. While most Hh pathway antagonists target a seven-transmembrane (7-TM) domain of Smoothened (Smo), components upstream and downstream of Smo are also being investigated as potential pharmaceutical targets^17^. The first example that a small molecule could effectively inhibit Hh pathway came from a natural product cyclopamine which binds Smo resulting in Hh pathway inactivation^18^.

GDC-0449 is the first FDA– approved Hh pathway inhibitor for the treatment of metastatic or locally advanced BCC^19^. Soon after GDC-0449, sonidegib was approved by the FDA as the second clinically prescribed Smo antagonist^20^. Dense desmoplasia in pancreatic ductal adenocarcinoma (PDAC) consists of primarily pancreatic stellate cell (PSC), fibroblasts and ECM proteins. It is driven by Hh signaling and limits the delivery of chemotherapy. GDC-0449 has shown the potential in reducing desmoplasia by targeting Smo. Despite the significant accomplishment in the development of Hh pathway inhibitors, the clinical use of GDC-0449 and sonidegib is severely restricted due to numerous side effects including constipation, diarrhea, decreased appetite, hair loss, muscle spasms, and tiredness^19, 21^. To overcome these obstacles, several Hh pathway inhibitors that target Smo have been developed. For instance, Castanedo *et al*., developed more potent second generation benzamide for inhibiting Hh pathway using *N*-[3-(2-pyridinyl) phenyl] benzamide scaffold common to the clinical lead compound GDC-0449^22^. Unfortunately, in addition to above restrictions, several challenges in the discovery and development process for small molecules targeting Hh pathway have been noted. For example, development of resistance to these drugs has become a major hurdle for their continued advancement^23–26^. Although treatment of MB patients with GDC-0449 demonstrated initial positive results, relapse was observed when a point mutation occurs in Smo rendering the patient insensitive to subsequent GDC-0449 treatment^23^. Moreover, regrowth of at least single BCC patient has been observed, when 21% of patients received continuous GDC-0449 treatment^27^. Also, aberrant expression of zinc finger transcription factor Gli, Phosphoinositide 3-kinase signaling pathway and low solubility of GDC-0449 limits its use^28, 29^. These bottlenecks of Hh pathway inhibitors have prompted an extensive search for novel inhibitors to function *via* mechanisms that will retain activity in the presence of pathway signaling resistant to current therapy.

Pancreatic cancer (PC) is one of the most aggressive and difficult cancers to treat. Limited success has been achieved in the therapeutic management of patients with this disease despite numerous research efforts. Hh pathway inhibitors are relatively new class of therapeutic agents that act by targeting Hh pathway protein components like Ptch, Smo, Gli and Sonic hedgehog (Shh). Together, they serve as exciting new prospects for PC treatment, both alone or as an adjuvant to the more traditional anticancer drugs. Therefore, development of better Hh pathway inhibitors with the understanding of the correlation between CSCs and EMT will help in cancer treatment. In this study, we have reported the use of structure-activity relationship (SAR) to design and synthesize novel GDC-0449 analogs with improved Hh pathway inhibition and anticancer properties. After screening of all the analogs, 2-chloro-*N* ^1^-[4-chloro-3-(2-pyridinyl) phenyl]-*N* ^4^,*N* ^4^-bis(2-pyridinylmethyl)-1,4-benzenedicarboxamide (MDB5) was found to be the best as evident from *in vitro* and *in vivo* studies.

## Results and Discussion

### Designing and Screening of GDC-0449 Analogs

PC is a leading cause of cancer-related mortality with a dismal 5–7% five-year survival rate. The current FDA-approved chemotherapeutic agent for PC is gemcitabine, which provides only symptomatic improvement in a lesser proportion of patients. New combination therapy FOLFIRINOX (fluorouracil [5-FU], leucovorin, irinotecan and oxaliplatin) showed improvement compared to gemcitabine alone; however, there was a significant rate of grade 3/4 toxicity in PC patients^30^.

**Figure 1:**
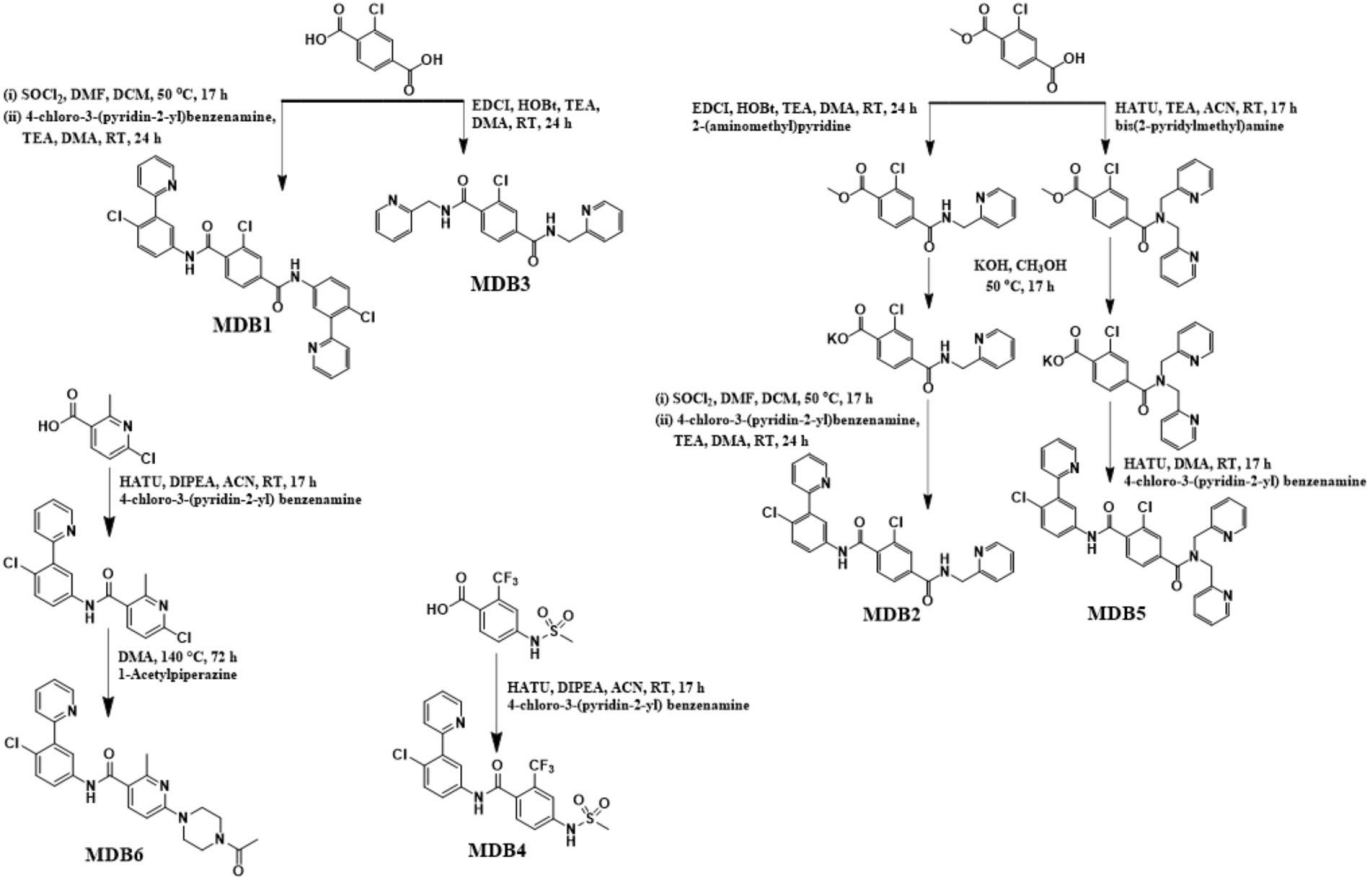
Schematic representation of GDC-0449 analogs synthesis. 2-chloro-*N* ^1^,*N* ^4^-bis[4-chloro-3-(2-pyridinyl)phenyl]-1,4-benzenedicarboxamide (MDB1), 2-chloro-*N* ^1^-[4-chloro-3-(2-pyridinyl)phenyl]-*N* ^4^-(2-pyridinylmethyl)-1,4-benzenedicarboxamide (MDB2), 2-chloro-*N* ^1^,*N* ^4^-bis(2-pyridinylmethyl)-1,4-benzenedicarboxamide (MDB3), *N*-[4-chloro-3-(2-pyridinyl)phenyl]-4-[(methylsulfonyl)amino]-2-(trifluoromethyl)benzamide (MDB4), 2-chloro-*N* ^1^-[4-chloro-3-(2-pyridinyl)phenyl]-*N* ^4^,*N* ^4^-bis(2-pyridinylmethyl)-1,4-benzenedicarboxamide (MDB5) and 6-(4-acetyl-1-piperazinyl)-*N*-[4-chloro-3-(2-pyridinyl)phenyl]-2-methyl-3-pyridinecarboxamide (MDB6) were synthesized using *N*-[3-(2-pyridinyl) phenyl]benzamide scaffold of GDC-9449.

Hh signaling plays a critical role in the formation of desmoplastic stroma; thus, promoting tumor growth and serve as a barrier to chemotherapy. This pathway is initiated when a family of Hh ligand (Desert, Indian or Shh) interacts with a cell surface transmembrane receptor Ptch (Ptch-1 and Ptch-2). This interaction relieves repression of Smo receptor and subsequently activate the downstream signaling. Activated Hh cascade permits nuclear localization of Gli family of transcription factors (Gli-1 and, Gli-2) that regulate the expression of genes associated with proliferation, angiogenesis, stemness and metastasis^2, 4, 5^. On the other hand, the tumor suppressor SuFu (suppressor of fused) negatively regulates Hh pathway by binding and sequestering Gli transcription factors in the cytoplasm^31–33^. Aberrant activation of Hh pathway has been shown to associate itself with a variety of human tumors including PDAC. Our earlier work on combining GDC-0449 with gemcitabine has shown synergistic downregulation of Hh pathway components inducing apoptosis^34^. Similar results were also seen in a panel of human PC cell lines including pancreatic CSC with GDC-0449^35^. Although Hh inhibitor GDC-0449 overcomes desmoplastic reaction by blocking oncogenic Smo involved in Hh signaling, its clinical use is restricted due to side effects and development of chemoresistance. Therefore, in this study, we considered GDC-0449 as a promising lead to further explore SAR around chlorobenzene (moiety 2) by using *N*-[3-(2-pyridinyl)phenyl]benzamide scaffold following synthetic scheme shown in Fig. 1. Since ortho-chloro group in moiety 2 is critical for GDC-0449 solubility, we focused our attention on moiety 3^22, 36^.

**Figure 2:**
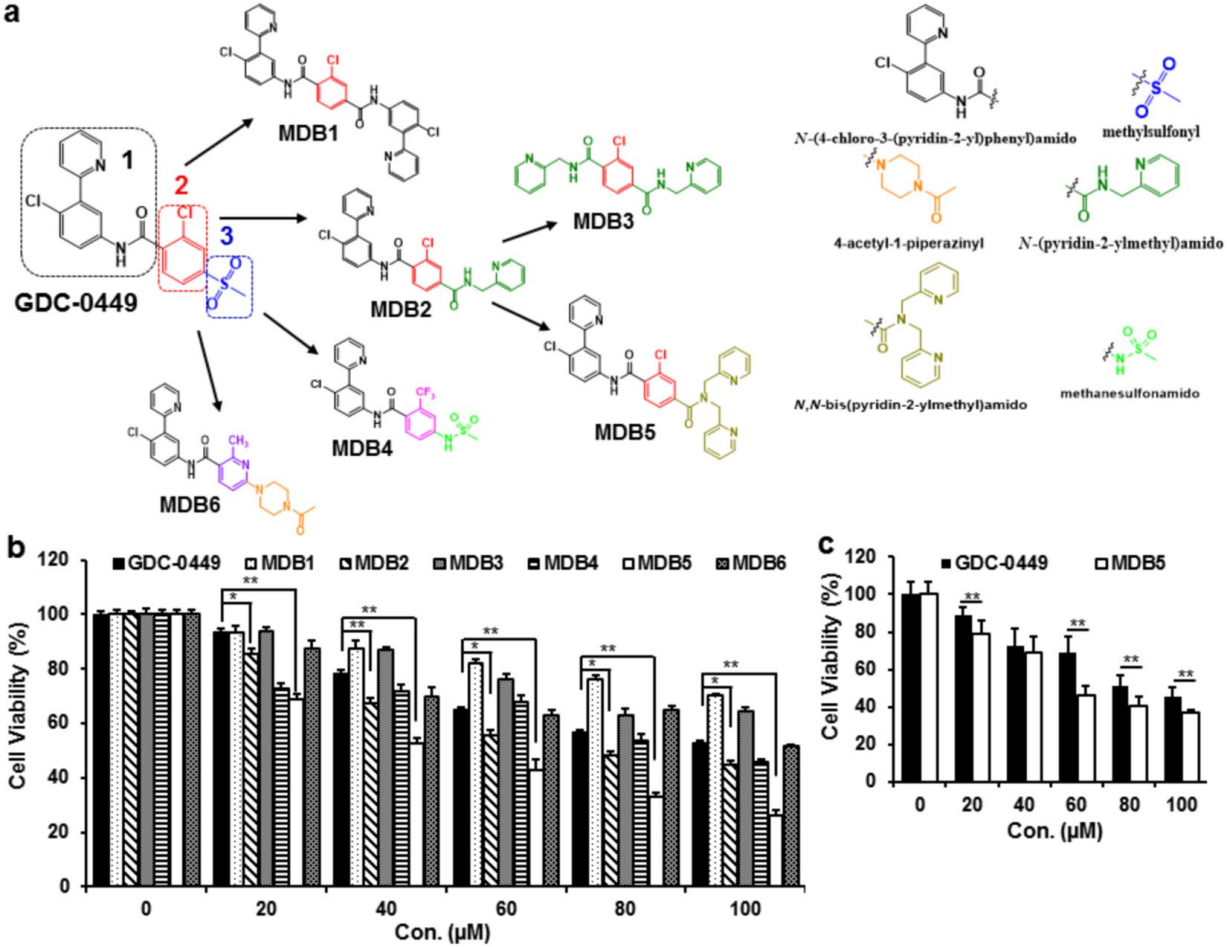
(**a**) Structure-activity relationship (SAR) to design GDC-0449 analogs. (**b**,**c**) Cell cytotoxicity assay of MIA PaCa-2 and PANC-1 cells treated with free drugs. Percentage of viable cells were estimated by MTT assay. Data is shown as the mean ± S.D. of three separate experiments (Student t-test; *p < *0.05*; **p < *0.001*; *n* = *3*).

Keeping moieties 1 and 2, we replaced methylsulfonyl group (moiety 3) with *N*-(4-chloro-3-(pyridin-2-yl)phenyl)amido group in GDC-0449 to generate MDB1 (Fig. 2a). The introduction of an additional *N*-(4-chloro-3-(pyridin-2-yl) phenyl)amido group in GDC-0449 did not show any enhanced cytotoxic effect in MIA PaCa-2 cells than GDC-0449 (Fig. 2b). This indicated that replacing moiety 3 with *N*-(4-chloro-3-(pyridin-2-yl) phenyl)amido group disrupted ligand-receptor interaction, thus leading to an undesirable activity (Table 1). A recent study shows that generation of 2-pyridyl biphenyl amides was potent in inhibiting Hh pathway as measured by Gli-1 transcript^22^. Therefore, we next replaced moiety 3 of GDC-0449 with *N*-(pyridin-2-ylmethyl)amido group to generate 2-pyridyl biphenyl amide 14f (abbreviated as MDB2) (Fig. 2a). The improved cell cytotoxicity profile of MDB2 in MIA PaCa-2 cells clearly suggest *N*-(pyridin-2-ylmethyl)amido group to be an important moiety for enhanced cytotoxic effect (Fig. 2b). To further exploit the benefit brought by *N*-(pyridin-2-ylmethyl)amido group, we replaced moiety 1 with *N*-(pyridin-2-ylmethyl)amido group in MDB2 to generate MDB3 (Fig. 2a). Although MDB3 contains two *N*-(pyridin-2-ylmethyl)amido groups at para positions of moiety 2, it did not exhibit better cytotoxic activity than GDC-0449 (Fig. 2b). In contrast, the presence of single *N*-(4-chloro-3-(pyridin-2-yl) phenyl)amido and single *N*-(pyridin-2-ylmethyl)amido groups around moiety 2 in MDB2 has better activity compared to GDC-0449. These results suggest that moiety 1 is not only necessary for solubility but also for bioactivity of GDC-0449. Therefore, we next increased the number of 2-pyridylmethyl group by attaching them to the same nitrogen atom in the amide-bond of MDB2, generating MDB5 (Fig. 2a). The substitution of moiety 3 of GDC-0449 with *N*,*N*-bis(pyridin-2-ylmethyl)amido group significantly improved cytotoxic activity in MIA PaCa-2 and PANC-1 cells (Fig. 2b and c). In the spirit of finding more potent analogs, we next replaced moieties 2 and 3 of GDC-0449 to generate MDB4 and MDB6 (Fig. 2a). In MDB4, we replaced –Cl with –CF_3_ in moiety 2 and methylsulfonyl in moiety 3 with methanesulfonamido group, whereas in MDB6, we replaced the chlorobenzene with methylpyridine in moiety 2 and moiety 3 with 4-acetyl-1-peperainyl group, respectively. Unfortunately, we did not observe any improved cytotoxic activity from MDB4 and MDB6 than GDC-0449 (Fig. 2b).

**Table 1:** IC_50_ (μM) in MIA PaCa-2 cells and docking scores (kcal/mol) of respective drugs in wild-type Smo, D473H and W281C mutants of Smo.

DRUGS	IC_50_ (μM)	Wild-type	D473H	W281C
GDC-0449	97.36	−9.41	−7.21	−9.10
MDB1	>100	−8.68	−9.08	−9.61
MDB2	79.73	−11.61	−9.88	−10.98
MDB3	>100	−8.90	−8.54	−9.01
MDB4	89.33	−9.91	−7.73	−9.32
MDB5	55.57	−12.84	−10.76	−11.52
MDB6	>100	−10.13	−8.33	−9.69

### Regulation of Hedgehog Signaling Through Smo Binding

**Figure 3:**
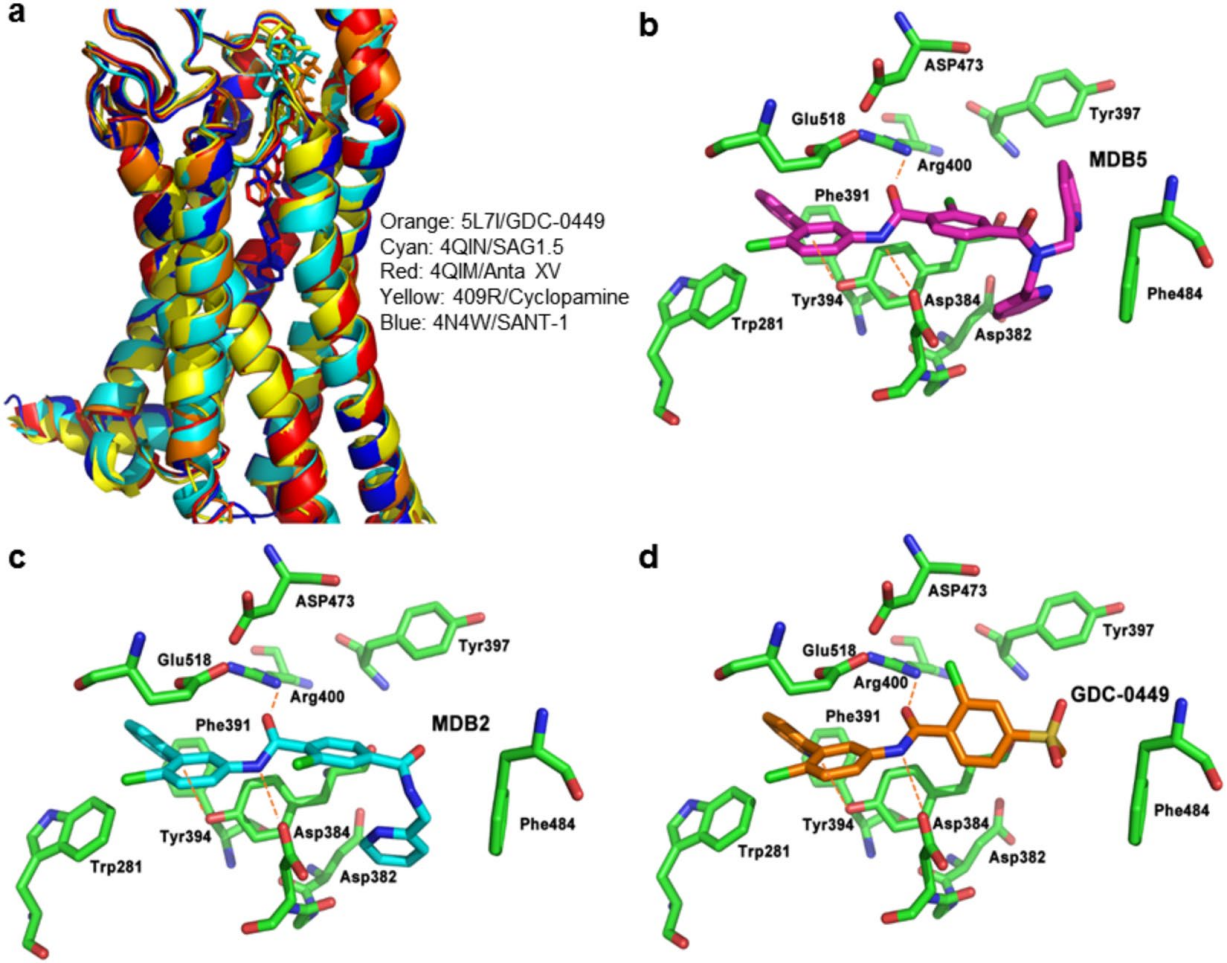
Molecular docking of GDC-0449 analogs in human Smo. (**a**) Binding pocket of Smo as defined by superposition of five crystal structures of human Smo (PDB ID: 5L7I, 4QIM, 4QIN, 4O9R and 4N4W). Structures and ligands were colored coded as noted. Smo/ligand interactions for MDB5 (**b**), MDB2 (**c**) and GDC-0449 (**d**). The hydrogen-bond interactions are depicted as orange dotted lines.

To test our hypothesis that an additional 2-pyridylmethyl group in MDB5 is necessary for the enhancement of Hh pathway inhibition and cell cytotoxicity, we performed molecular docking studies with human Smo containing 7-TM and extracellular cysteine-rich ligand-binding domains^37^. Followed by Smo crystal structures (PDB ID: 5L7I, 4QIM, 4QIN, 4O9R and 4N4W) alignment and docking of reference compounds, we observed GDC-0449, Anta XV (2-(6-(4-(4-benzylphthalazin-1-yl)piperazin-1-yl)pyridin-3-yl)propan-2-ol), SAG1.5 (3-chloro-4,7-difluoro-*N*-[trans-4-(methylamino)cyclohexyl]-*N*-[[3-(4-pyridinyl)phenyl]methyl]-1-benzothiophene-2-carboxamide) and cyclopamine to bind in the similar pocket closer to the upper opening of 7-TM domain. However, SANT-1 (N-[(1E)-(3,5-dimethyl-1-phenyl-1-hpyrazol-4-yl)methylidene]-4-(phenylmethyl)-1-piperazinamine) was found to be a deep-pocket binder (Fig. 3a)^38^. Further, the docking of GDC-0449 and its analogs to Smo yielded MDB5 as the strongest binder with the docking score of −12.84 kcal/mol, the lowest among all (Fig. 3b) (Table 1). Inspection of the binding interactions between stronger binders (MDB2 and MDB5) versus GDC-0449 showed some shared and distinct interactions for each compound. Where all of them shared similar hydrogen-bonding with Asp384 and Arg400 to amide-bond, Tyr394 shared to pyridine nitrogen atom (Fig. 3b–d). These common interactions suggest the importance of pyridine ring (ortho to the chlorine atom) and amide-bond (para to the chlorine atom) in moiety 1. The importance of moiety 1 was validated by our current study, which shows that, replacement of moiety 1 with a pyridine ring significantly reduced the activity of MDB3. In addition to the common interactions, MDB2 and MDB5 possess different interactions due to their distinct structural features. For instance, the two pyridinylmethyl groups on MDB5 allows further interactions with Asp382, Tyr397 and Phe484 (Fig. 3b). For MDB2, the mono pyridinylmethyl group forms interactions with Asp382 and Phe484, thus making MDB2 a weaker binder than MDB5 due to the missing interactions with Tyr397. However, the additional interactions with Smo still make MDB2, a stronger binder than GDC-0449 (Fig. 3b,c and d). Therefore, the pyridinylmethyl groups (mono or di-substituent) would need to be kept in future structural optimization. Caution needs to be taken that the space composed of Asp382, Tyr397 and Phe484 is limited allowing certain structural modifications. Structural modifications that lead to large groups would weaken the binding. For instance, the additional moiety 1 of MDB1, however, made it too bulky for 7-TM domain of Smo, thus caused steric repulsion with Phe484 leading to much weaker activity. Pharmacodynamically, MDB1 was predicted to have a similar binding score as MDB3. However, the much larger hydrophobicity of MDB1 (logP 7) makes it more difficult to absorb and becomes more disruptive to Smo binding. Both pharmacodynamics and pharmacokinetic properties of MDB1 contributed to weaker docking scores and observed weaker activities (Table 1).

**Figure 4:**
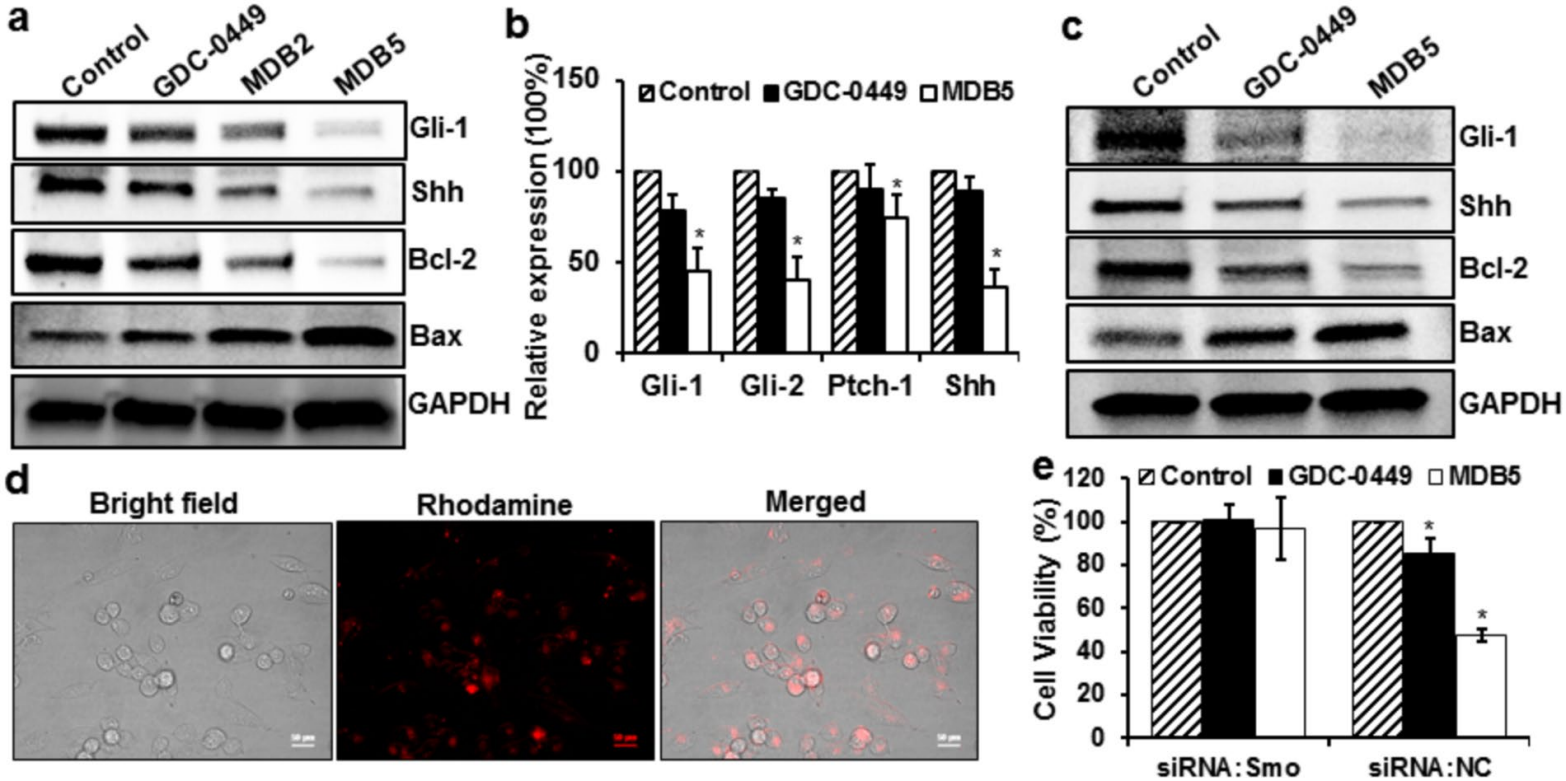
Effect of MDB5 on Hh pathway and target genes. (**a**) Western blot analysis for Gli-1, Shh, Bcl-2, Bax and GAPDH of MIA PaCa-2 cells treated with GDC-0449 and MDB5. (**b**) Real-time RT-PCR analysis of Gli-1, Gli-2, Ptch-1 and Shh of MIA PaCa-2 cells treated with GDC-0449 and MDB5. **(c)** Western blot analysis for Gli-1, Shh, Bcl-2, Bax and GAPDH of PANC-1 cells treated with GDC-0449 and MDB5. (**d**) Transfection of labeled oligonucleotide using Lipofectamine® RNAiMAX in MIA PaCa-2 cells. (**e**) Cell cytotoxicity assay of GDC-0449 and MDB5 in Smo knockdown MIA PaCa-2 cells. Percentage of viable MIA PaCa-2 cells was estimated by MTT assay. Data is shown as mean ± SD of three separate experiments (Student t-test; *p < *0.05*; *n* = *3*). Cropped Western blots are shown. Full-length Western blots are included in the Supplementary Information (Fig. S14).

Human PC cells (AsPC-1, PANC-1, and MIA-PaCa-2) and pancreatic CSC express differential level of Hh pathway components (Ptch, Smo, Gli and Shh) and mesenchymal markers^35, 39^. Under physiological condition, Hh pathway inhibitors target these components, disturbing the downstream signaling. Our earlier study has shown that the combination of GDC-0449 and gemcitabine has distinct effects on MIA PaCa-2 cells compared to others^34^. Similar effects were seen when GDC-0449 was combined with miR-let7b on MIA PaCa-2 cells, as the aberrant expression of miRNAs is critical for cancer initiation and progression^28, 40^. Since MIA PaCa-2 and PANC-1 cells express profound level of Hh pathway components^35^, we observed a significant reduction of Gli-1 and Shh proteins in these cells after MDB5 treatment (Fig. 4a and c)^22^. Similarly, a decrease in Gli-1, Gli-2, Ptch and Shh transcripts were also evident in MDB5 treated MIA PaCa-2 cells compared to GDC-0449 (Fig. 4b). Since transcription factors Gli is known to play a regulatory role in cell cycle and apoptosis; our observations suggest MDB5 act on Hh pathway with much more Smo binding capacity than GDC-0449.

As GDC-0449 is Smo antagonist; we further sought to validate our molecular simulation in Smo knockdown cells using Smo specific siRNA. As shown in Fig. 4e, GDC-0449 and MDB5 were unable to show cytotoxic effect when MIA PaCa-2 cells were transfected with ON-TARGETplus Smo specific siRNA before drug treatment. However, a similar cytotoxic effect was observed when the cells were transfected with non-targeting siRNA before drug treatment (Figs 2b and 4d,e). These observations clearly suggest that MDB5 acts via Smo and share a common pathway like its parent compound GDC-0449, thus did not show any cytotoxic effect after inhibition of Smo expression like GDC-0449.

Although GDC-0449 overcomes impenetrable desmoplastic stroma, its clinical use is limited mainly due to the development of mutations in Smo leading to chemoresistance. Smo mutant D473H has shown to rendered resistance to GDC-0449^41^ by reducing its binding to Smo with 100-fold compared to the wild-type^42^. Similarly, mutant W281C decreases binding affinity of GDC-0449 to Smo by 40-fold as measured by Kd^43^. Therefore, to evaluate the binding of our analogs to Smo mutants, we generated Smo mutants computationally by mutating D473 into His (D473H) and W281 into Cys (W281C), followed by docking of MDB1–6 and GDC-0449. The binding scores of GDC-0449 to D473H and W281C dropped from −9.41 kcal/mol in wild-type to −7.21 and −9.10 kcal/mol respectively, which is in good agreement with the observation that D473H and W281C significantly reduced GDC-0449 binding. In contrast, the binding scores of MDB5 to D473H and W281C increases to −10.76 and −11.52 kcal/mol respectively, compared to GDC-0449 showing its tighter binding affinity than GDC-0449 (Table 1). These evidence suggested MDB5 may still be sensitive to D473H and W281C mutants due to the lower docking scores compared to GDC-0449.

### Regulation of Apoptosis and Cell Cycle Arrest

**Figure 5:**
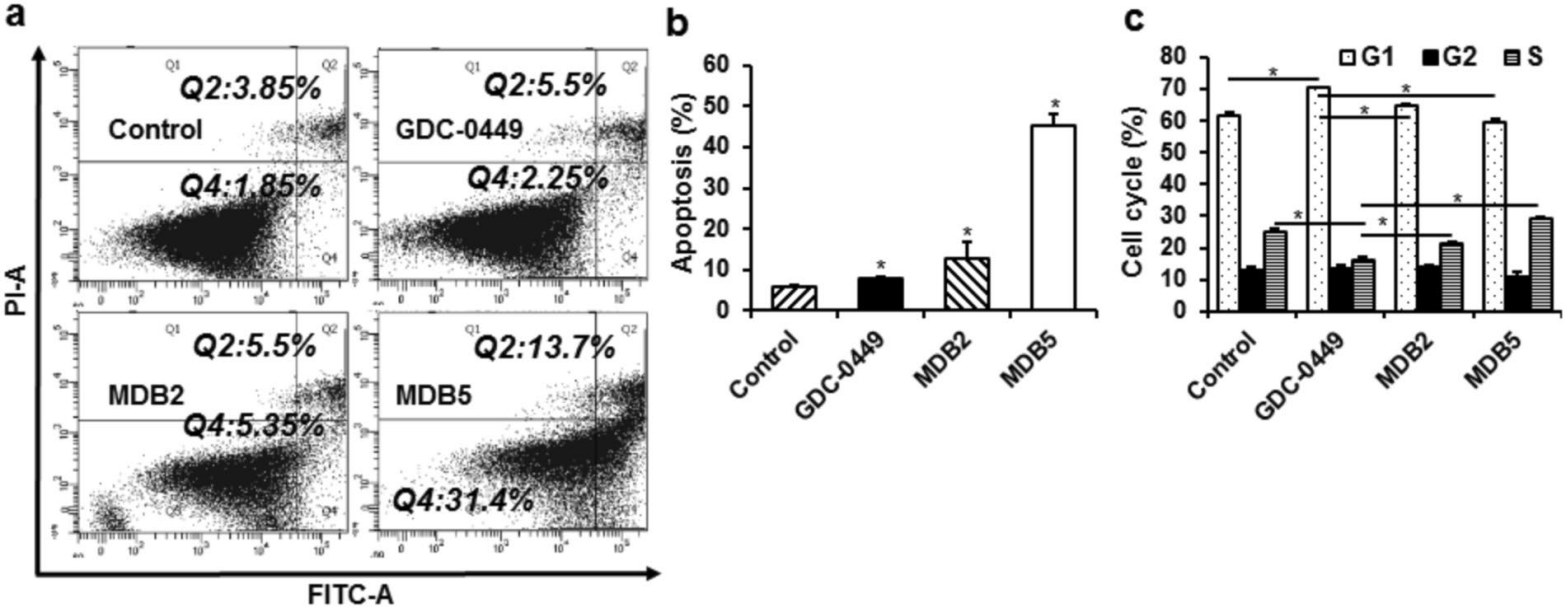
(**a**) Induction of apoptosis by MDB5 in MIA PaCa-2 cells. (**b**) Graphical representation of total percentage of apoptosis induced by GDC-0449 and MDB5 (Student t-test; *p < *0.05* vs. control). (**c**) Analysis of cell cycle arrest in MIA PaCa-2 cells. The graphical representation of percentage of cells in G0/G1, G2/M and S-phase of the cell cycle (Student t-test; *p < *0.05*, *n* = *3*).

Apoptosis plays a major role in maintaining the homeostasis of normal cells. Cellular shrinkage and DNA fragmentation at the molecular level are characteristics acquired during apoptosis^44^. Therefore, we performed flow cytometric analysis using MIA PaCa-2 cells, where the total apoptotic cells populations significantly increased from 7.75% in GDC-0449 to 45.1% in MDB5, whereas 10.85% and 5.7% of apoptotic cells were seen in MDB2 and control treated samples, respectively (Fig. 5a and b). Since Bax pro-apoptotic protein accelerates the cell death in response to apoptotic stimuli, we next measured the level of Bax as an indicator of apoptosis. In Fig. 4a and c, we observed increased Bax expression in MIA PaCa-2 and PANC-1 cells treated with MDB5 compared to GDC-0449. In contrast, the anti-apoptotic protein Bcl-2 was significantly downregulated in cells induced by MDB5 compared to GDC-0449. As Bcl-2 is overexpressed in various cancers and contribute to treatment resistance by inhibiting chemotherapy-induced apoptosis^45, 46^, our observations provide a reasonable mechanistic explanation of the observed cell death induction.

Based on our molecular simulation and Smo knockdown study, it is clear that MDB5 exerts antiproliferative effect by targeting and destabilizing Smo. Therefore, we next performed cell cycle analysis by flow cytometry to evaluate the effect of MDB5 on cell cycle arrest. We observed a significant increment in S-phase from 16.15% in GDC-0449 to 21.25% and 29.27% in MDB2 and MDB5 treated MIA PaCa-2 cells, respectively. This result confirmed that the anticancer effect of MDB5 is through the arrest of cell cycle in S-phase and subsequent activation of intrinsic apoptotic cellular machinery. In contrast, a significant reduction of cells in G1-phase was evident in MDB2 and MDB5 treated cells, respectively (Fig. 5c).

### Role of GDC-0449 Analogs on Pancreatic CSC, Cell Migration and Tumorigenicity

**Figure 6:**
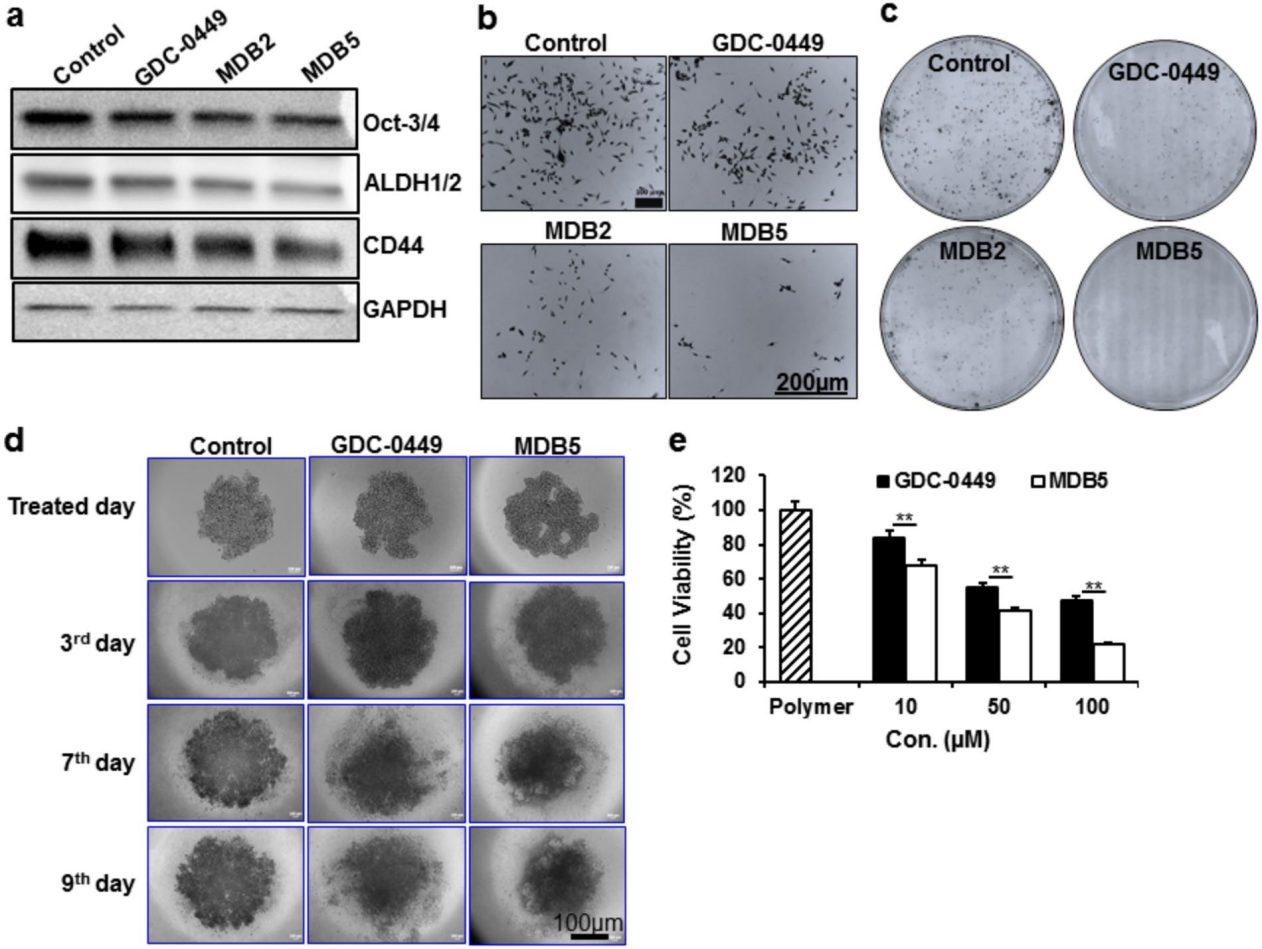
(**a**) Western blot analysis of pancreatic CSC markers in MIA PaCa-2 cells after GDC-0449 and MDB5 treatment. (**b**) Effect of GDC-0449 and MDB5 on MIA PaCa-2 cells motility. **(c)** Effect of GDC-0449 and MDB5 on colony formation of MIA PaCa-2 cells. (**d**) Effect of GDC-0449 and MDB5 on 3D spheroid of MIA PaCa-2 cells. (**e**) Cell cytotoxicity assay of MIA PaCa-2 cells treated with GDC-0449 and MDB5 containing nanoparticles. Percentage of viable MIA PaCa-2 cells was estimated by MTT assay. Data are shown as the mean ± S.D. of three separate experiments (Student t-test; *p < *0.05*; **p < *0.001*; *n* = *3*). Cropped Western blots are shown. Full-length Western blots are included in the Supplementary Information (Fig. S14).

There is a growing attention that CSCs are often related to metastasis, chemoresistance and EMT phenotype^47–49^. The resistance to chemotherapy of CSCs prevents the eradication of cancer and presents the looming threat of recurrence. The growing evidence suggest the existence of CSCs in PC overexpressing aldehyde dehydrogenases (ALDH), CD44 and Oct-3/4 including others^50–57^. Studies show that ALDH-high cells comprise more sub-population of cells in human PC that are tumorigenic and capable of producing tumors at very low numbers^58^. Recently, Singh *et al*. show that GDC-0499 inhibits pancreatic CSC cell viability and induces apoptosis through caspase-3 activation and poly (ADP-ribose) polymerase (PARP) cleavage^35^. Therefore, we examined the expression of ALDH1, CD44 and Oct-3/4 to further confirm the effect of MDB5 on pancreatic CSC. As shown in Fig. 6a, a significant reduction in ALDH1, CD44 and Oct-3/4 was observed in MIA PaCa-2 cells indicating the ability of MDB5 to kill pancreatic CSC effectively than GDC-0449. Moreover, a significant reduction of Bcl-2 by MDB5 in MIA PaCa-2 and PANC-1 cells compared to GDC-0449 provides an additional reasonable explanation of MDB5 effect on pancreatic CSC (Fig. 4a and c). Likewise, a significant decrease of Gli-1, Gli-2, Ptch-1 and Shh transcripts as supported by Singh *et al*.^35^ studies provide an additional explanation that MDB5 is more efficient in inhibiting pancreatic CSC compared to GDC-0449 (Fig. 4b).

During EMT, the cells lose their epithelial features including loss of sheet-like architecture and polarity resulting in migration, invasion and increased tumorigenic potential^59–61^. A recent study shows that the activated Hh pathway promoting tumorigenicity and stemness via EMT, attenuate cell motility, invasiveness, and clonogenic potential of bladder and non-small cell lung cancers after GDC-0449 treatment^62, 63^. As EMT is relevant to the acquisition and maintenance of stem cell-like characteristics and is sufficient to endow differentiated normal and cancer cells with stem cell properties, we next checked the effect of MDB5 on cell migration and tumorigenic potential of MIA PaCa-2 cells. Supporting our other results, we observed a significant decrease in the number of migratory cells in MDB5 treated highly invasive and metastatic PC cells compared to GDC-0449 and MDB2 (Fig. 6b). Additionally, MDB5 also reduces the tumorigenic potential of MIA PaCa-2 cells significantly compared to GDC-0449 and MDB2, respectively (Fig. 6c). These evidence supported MDB5 to be more potent in reducing pancreatic CSC, invasive and tumorigenic potential of PC affecting EMT phenotypes.

### Effects of MDB5 on 3D Spheroids and *In Vivo* Tumor Regression

Recently, increasing numbers of cell culture experiments with 3D spheroids presented better correlating results *in vivo* than traditional 2D culture systems. It became apparent that 3D cultures are more resistant to chemo-radiotherapy than their 2D counterparts. Compared to 2D culture models, 3D spheroids can accurately mimic features of solid tumors, such as their spatial architecture, physiological responses, secretion of soluble mediators, production of extracellular matrix, gene expression patterns and drug resistance mechanisms. These unique characteristics highlight the potential of 3D spheroids and offer a simple and highly reproducible model for *in vitro* drug screening^64, 65^. Most drugs that pass preclinical tests successfully, fail miserably in the patient due to traditional 2D culture for drug screening. Therefore, we developed 3D spheroids of MIA PaCa-2 cells, and as evident from our 2D culture MTT assay (Fig. 2b), we observed a significant reduction in spheroid size in MDB5 treated group compared to GDC-0449 (Fig. 6d). This result further supports MDB5 as more potent analog than GDC-0449.

**Figure 7:**
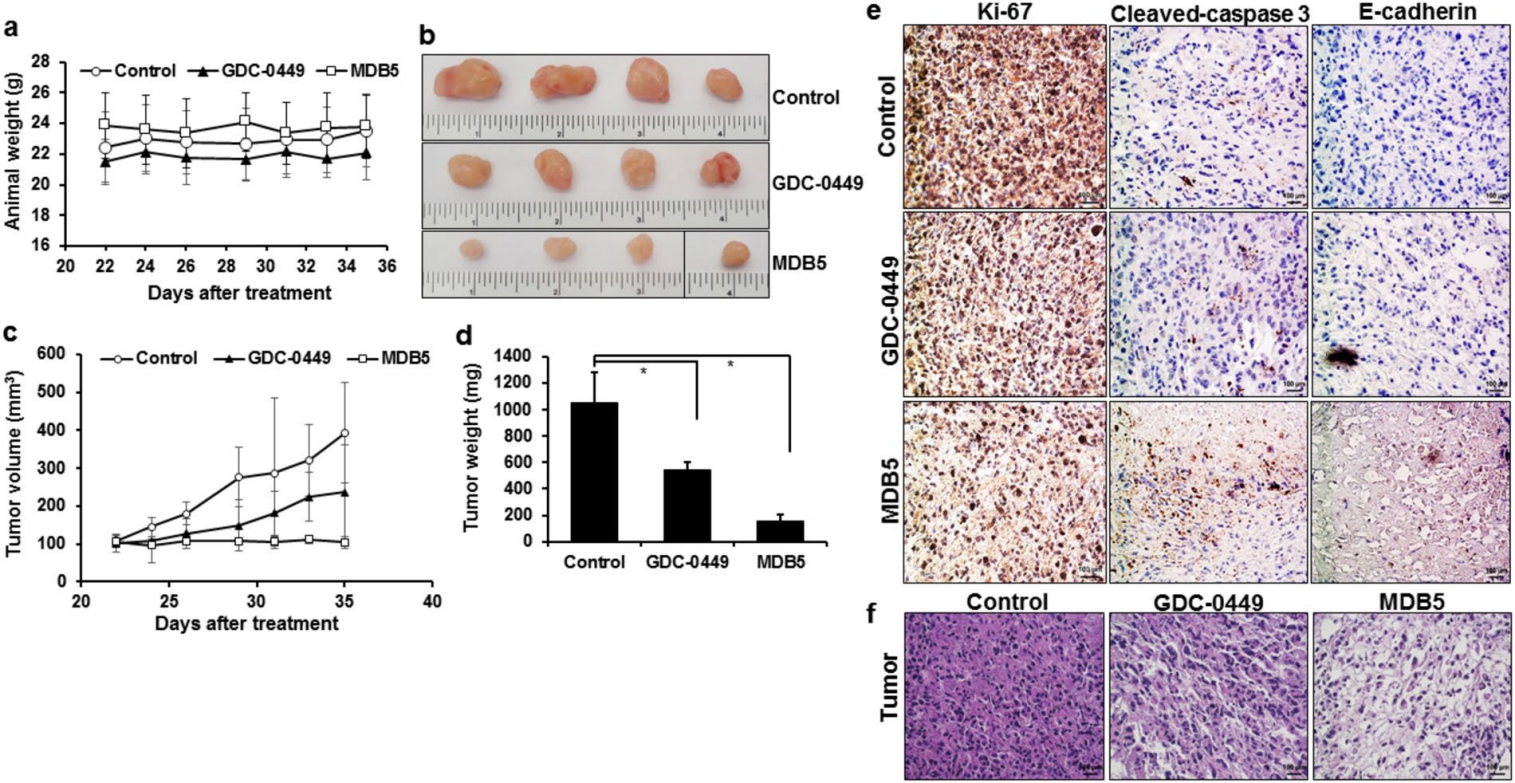
*In vivo* efficacy of nanoparticles carrying GDC-0449 and MDB5 in tumor bearing mice. (**a**) Animals body weight during treatment. (**b**) Representative tumor size of various treatment groups. **(c)** Tumor volume. (**d**) Tumor weight. Data are expressed as the mean ± S.E. (*n* = *5*) (Student t-test; *p < *0.05*, *n* = *5*). (**e**) Immunohistochemical analysis of tumor samples by Ki-67, Cleaved-caspase 3 and E-cadherin staining. Scale bar, 100 μm. (**f**) H&E staining of tumor samples. Scale bar, 100 μm.

A suitable formulation of drugs is an essential step in drug development. However, most drugs are usually developed for intravenous use, despite drawbacks like thrombosis and extravasation, and risk of intravenous-catheter-related infection^66^. Additionally, solubility is a specific demand for intravenous administration of drugs, even for poor water-soluble new generation of chemotherapeutic agents. Classical solubilizing approaches including the use of Tween 80 and/or cremophor EL significantly increased the solubility of hydrophobic drugs and protect them from premature degradation within the systemic circulation. However, these solubilizing agents are usually associated with liver and kidney toxicity, hemolysis, acute hypersensitivity reaction and peripheral neuropathies^67^. Since GDC-0449 analogs are hydrophobic in nature, the use of solubilizing agents and surfactants for *in vivo* delivery may cause organ and system toxicity. In our earlier study, bicalutamide-loaded PEG-b-P(CB-co-LA) micelles^68^ and LY293-loaded mPEG-b-P(CB-co-LA) nanoparticles^69^ showed exceptional efficacy in inhibiting human prostate cancer and melanoma, respectively. Also, mPEG-b-P(CB-co-LA) nanoparticles provided an excellent platform to co-deliver different poorly soluble drugs like GDC-0449 and PPAR-γ agonist, rosiglitazone, respectively to treat liver fibrosis^70^. As nanoparticles improve the pharmacokinetic profiles, increases solubility and drug loading with reduced toxicity, in the present study, we used mPEG-b-P(CB-co-LA) nanoparticles for *in vivo* delivery of MDB5. To continue this endeavor, we formulated biodegradable polymeric nanoparticles to evaluate the efficacy of GDC-0449 and MDB5 in subcutaneous pancreatic tumor bearing NSG mice generated using MIA PaCa-2 cells. Nanoparticles were prepared using mPEG-b-p(CB-b-LA) copolymer with the mean particle size of 113.6 ± 5 nm and 132.6 ± 2 nm for GDC-0449 and MDB5, respectively (Fig. S13). Following drug loading of 5% (Fig. S13), MIAPaCa-2 cells treated with nanoparticles containing MDB5 showed similar inhibition of cell viability as of free drugs (Fig. 2b) in dose-dependent manner (Fig. 6e). Following *in vitro* analysis, the mice treated with nanoparticles containing GDC-0449 and MDB5 showed a significant reduction in tumor growth compared to control (392.22 ± 52.43 mm^3^). There was much more reduction in tumor growth in the mice treated with nanoparticles carrying MDB5 (103.56 ± 16.25 mm^3^) compared to mice treated with nanoparticles carrying GDC-0449 (237.25 ± 42.76 mm^3^). Also, no significant changes in body weight of the animals were observed during the study indicating tolerance to the doses of GDC-0449 and MDB5 (Fig. 7a–d).

**Figure 8:**
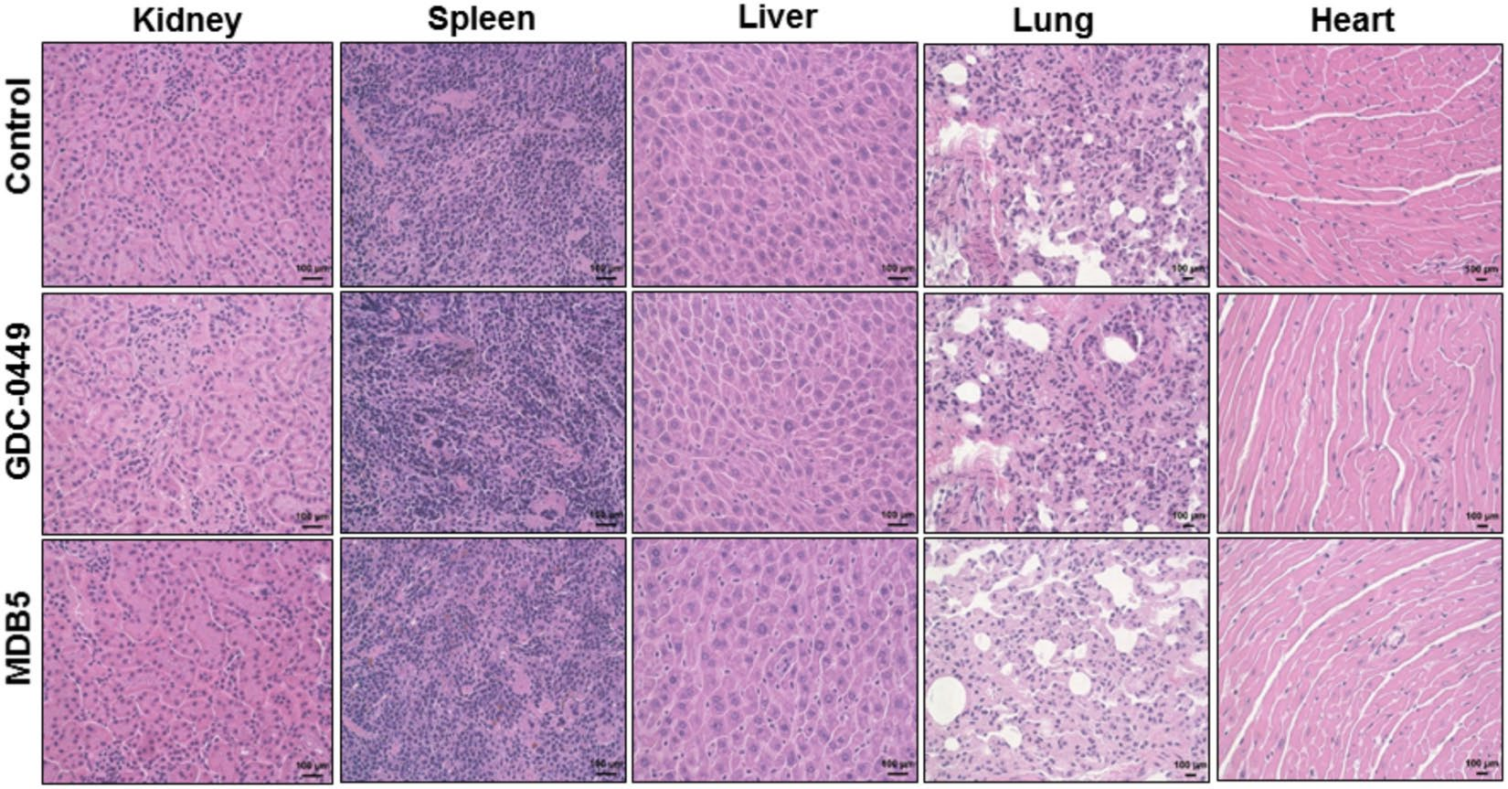
Immunohistochemical analysis of major organs (kidney, spleen, liver, lung and heart) by H&E staining. Scale bar, 100 µM.

Further, to elucidate the superior anticancer effect of MDB5, we performed immunohistochemical analysis of proliferation (Ki-67), apoptosis (cleaved caspase-3) and EMT (E-cadherin) markers. As shown in Fig. 7e, nanoparticles containing MDB5-treated mice displayed a lesser level of Ki-67 but a higher level of cleaved caspase-3 and E-cadherin compared to nanoparticles containing GDC-0449 and control treated tumors, respectively (Fig. 7e). Additionally, histological staining of mice treated with PBS (control) showed loss of cellular architecture with hypercellularity, whereas mice treated with nanoparticles containing MDB5 showed a significant pyknosis, compared to nanoparticles containing GDC-0449 treated tumors (Fig. 7f). Also, histological staining of major organs (kidney, spleen, liver, lung and heart) did not show any pathological changes after treatment in all the groups (Fig. 8). These promising results obtained from treated groups of mice further confirmed MDB5 to be effective in the inhibition Hh pathway and most potent anticancer agent with negligible systemic toxicity.

Additionally, at tested dose of 20 and 40 mg/kg of MDB5, we did not observe any significant changes in biochemical parameters of such as alanine aminotransferase (ALT) and total bilirubin (TBIL) in treated animals compared to the control group confirming negligible hepatic injury (Table S1).

## Conclusions

Smo is a major component of Hh signaling which play a major role in the development and metastasis of PC. In the spirit of finding more potent GDC-0449 analogs, in this study, we designed and synthesized six analogs (MDB1-6). Among these analogs, MDB5 showed better Hh pathway inhibition and anticancer activities than GDC-0449. Significant upregulation of Bax and downregulation of Bcl-2, Gli-1, Shh, ALDH1, CD44 and Oct-3/4 proteins by MDB5 confirms its better Hh pathway and pancreatic CSC inhibition properties compared to GDC-0449. Our molecular docking suggested that MDB5 binds with extra interactions in 7-TM domain of Smo due to the presence of an additional 2-pyridylmethyl group and thus became a stronger binder than GDC-0449. Additionally, our studies agreed with the experimental observation that mutants D473H and W281C led to decreased binding affinity toward GDC-0449 and that our best compound MDB5 might still be sensitive to mutants D473H and W281C, which would be possible due extra binding interactions observed MDB5. Compared to GDC-0449, MDB5 significantly inhibited the tumor growth without any systemic toxicity. These evidence highlight the enhanced Hh pathway inhibition and anticancer properties of MDB5.

Hh signaling is not only limited to cancer but also to other diseases. In liver fibrosis, secretion of growth factors and Hh ligands by hepatic parenchyma upon repeated insults results in transdifferentiation of quiescent hepatic stellate cells (HSCs) into active myofibroblasts which secrete excessive amounts of extracellular matrix (ECM) proteins. Therefore, Hh pathway inhibitors can play a major role in treating liver fibrosis by inhibiting several pro-fibrotic genes^29, 70, 71^. In conclusion, further exploration of GDC-0449 analog can leave a platform for developing more potent Hh inhibitors for treating diseases related to Hh signaling.

## Methods

### Chemicals and Reagents

GDC-0449 (Vismodegib) was purchased from LC Laboratories (Woburn, MA). TaqMan reverse transcription reagent kit was purchased from Life Technologies (Grand Island, NY). Radioimmunoprecipitation assay (RIPA) buffer and SYBR green-1 were purchased from Roche (Indianapolis, IN). All other reagents including 3-(4,5-dimethylthiazol-2-yl)-2,5-diphenyltetrazolium bromide (MTT), 2-chloroterephthalic acid (C_8_H_5_ClO_4_), thionyl chloride (SOCl_2_), dimethylformamide (DMF), dichloromethane (CH_2_Cl_2_), 4-chloro-3-(pyridin-2-yl)benzenamine (C_11_H_9_ClN_2_), triethanolamine (TEA), dimethylacetamide (DMA), acetonitrile (ACN), 3-chloro-4-(methoxycarbonyl) benzoic acid (C_9_H_7_ClO_4_), 2-(aminomethyl)pyridine (C_6_H_8_N_2_), hydroxybenzotriazole (HOBt), 1-ethyl-3-(3-dimethylaminopropyl)carbodiimide (EDCI), ethyl acetate (EtOAc), magnesium sulfate (MgSO_4_), potassium hydroxide (KOH), methanol (CH_3_OH), dimethyl ether (DME), *N*,*N*-diisopropylethylamine (DIPEA), 1-[bis(dimethylamino)methylene]-1*H*-1,2,3-triazolo[4,5-*b*]pyridinium 3-oxid hexafluorophosphate) (HATU), bis(2-pyridylmethyl)amine (C_12_H_13_N_3_), 6-chloro-2-methylpyridine-3-carboxylic acid (C_7_H_6_ClNO_2_), 1-acetylpiperazine (C_6_H_12_N_2_O), 4-(methanesulfonylamino)-2-(trifluoromethyl)benzoic acid were purchased from Sigma-Aldrich (St. Louis, MO).

### Synthesis of GDC-0449 analogs

Figures S1–S12 shows the synthetic scheme and structural characterization of MDB1, MDB2, MDB3, MDB4 and MDB6.

#### 2-chloro-*N*^1^,*N*^4^-bis[4-chloro-3-(2-pyridinyl)phenyl]-1,4-benzenedicarboxamide (MDB1)

MDB1 was synthesized by heating a mixture of C_8_H_5_ClO_4_ (506 mg, 2.52 mmol), SOCl_2_ (2 mL) and DMF (a drop) in CH_2_Cl_2_ (5 mL) at 50 °C for 17 h. The reaction was cooled to room temperature and concentrated. The residue was diluted with CH_2_Cl_2_ (10 mL) and concentrated to dryness. To the residue was added a solution of C_11_H_9_ClN_2_ (1.03 g, 5.04 mmol) and TEA (1.0 g, 10 mmol) in DMA (10 mL). The mixture was stirred at room temperature for 24 h and then quenched with water (50 mL). The resulting precipitate was collected by filtration, rinsed with ACN (20 mL), and dried to give the diamide MDB1 (1.07 g, 74%) as a white solid. Mp 130–132 °C. ^1^H NMR (500 MHz, DMSO-*d*_6_) 7.43–7.47 (m, 2H), 7.58–7.61 (m, 2H), 7.69–7.73 (m, 2H), 7.77–7.82 (m, 2H), 7.91–7.97 (m, 3H), 8.03–8.09 (m, 3H), 8.19 (s, 1H), 8.70–8.73 (m, 2H), 10.65 (s, 1H), 10.88 (s, 1H); ^13^C NMR (125.7 MHz, DMSO-*d*_6_) 121.13, 121.79, 122.60, 123.16, 123.46, 124.69, 125.80, 126.91, 128.88, 129.29, 130.34, 130.39, 130.53, 136.60, 137.05, 137.98, 138.11, 139.16, 139.31, 139.39, 149.65, 149.67, 156.02, 163.69, 164.66.

#### 2-chloro-*N*^1^-[4-chloro-3-(2-pyridinyl)phenyl]-*N*^4^-(2-pyridinylmethyl)-1,4-benzenedicarboxamide (MDB2)

MDB2 was synthesized in two steps. In first step, to a mixture of C_9_H_7_ClO_4_ (1.07 g, 5 mmol), C_6_H_8_N_2_ (810 mg, 7.5 mmol), HOBt (675 mg, 5 mmol), and TEA (1.01 g, 10 mmol) in DMA (20 mL) was added EDCI (1.44 g, 7.5 mmol). The mixture was stirred at room temperature for 17 h and then quenched with water (100 mL). The mixture was extracted with EtOAc (3 × 50 mL). The organic layers were washed with water (50 mL) and brine (50 mL), dried over MgSO_4_, filtered and concentrated. In second step, a mixture of the crude amide and KOH (561 mg, 10 mmol) in CH_3_OH (50 mL) was heated at 50 °C for 17 h and cooled to room temperature. After removal of the solvent, the residue was purified by crystallization from CH_2_Cl_2_ (20 mL) to give the desired potassium salt MDB2 as a white solid (1.80 g). Step 3. A mixture of the potassium salt (0.90 g), SOCl_2_ (1 mL) and DMF (a drop) in DME (5 mL) was heated at 60 °C for 17 h. The reaction was cooled to room temperature and concentrated. The residue was diluted with DME (5 mL) and concentrated to dryness. To the residue was added a solution of C_11_H_9_ClN_2_ (410 mg, 2 mmol) and TEA (404 mg, 4 mmol) in DMA (10 mL). The mixture was stirred at room temperature for 24 h and then quenched with brine (50 mL). After the solvent was decanted, the residue was purified by crystallization from water to afford the desired diamide MDB2 (513 mg, 43%) as a white solid. Mp 94–96 °C. ^1^H NMR (500 MHz, DMSO-*d*_6_) 4.61 (d, *J* = 5.5 Hz, 2H), 7.25–7.31 (m, 1H), 7.35 (d, *J* = 8.0 Hz, 1H), 7.42–7.47 (m, 1H), 7.58 (d, *J* = 8.5 Hz, 1H), 7.70 (d, *J* = 8.0 Hz, 1H), 7.71–7.81 (m, 3H), 7.91–7.95 (m, 1H), 7.99 (dd, *J* = 8.0, 1.0 Hz, 1H), 8.04 (d, *J* = 2.5 Hz, 1H), 8.10 (s, 1H), 8.53 (d, *J* = 4.0 Hz, 1H), 8.72 (d, *J* = 4.5 Hz, 1H), 9.37 (t, *J* = 5.5 Hz, 1H), 10.85 (s, 1H); ^13^C NMR (125.7 MHz, DMSO-*d*_6_) 45.02, 121.12, 121.22, 122.35, 122.59, 123.15, 124.69, 125.79, 126.41, 128.57, 129.26, 130.34, 130.51, 136.59, 136.85, 136.93, 137.99, 138.95, 139.37, 149.08, 149.66, 156.02, 158.51, 164.60, 164.73.

#### 2-chloro-*N*^1^,*N*^4^-bis(2-pyridinylmethyl)-1,4-benzenedicarboxamide (MDB3)

MDB3 was synthesized as follows. To a mixture of C_8_H_5_ClO_4_ (401 mg, 2 mmol) and C_6_H_8_N_2_ (649 mg, 6 mmol), HOBt (270 mg, 2 mmol), and TEA (606 mg, 6 mmol) in DMA (10 mL) was added EDCI (1.15 g, 6 mmol). The mixture was stirred at room temperature for 24 h and then quenched with water (100 mL). The mixture was extracted with EtOAc (3 × 50 mL). The combined organic layers were washed with water (50 mL) and brine (50 mL), dried over MgSO_4_, filtered, and concentrated. The crude product was purified by crystallization from ACN to give the desired product (253 mg, 33%) as a white solid. Mp 143–145 °C. ^1^H NMR (500 MHz, DMSO-*d*_6_) 4.57 (d, *J* = 6.0 Hz, 2H), 4.59 (d, *J* = 6.0 Hz, 2H), 7.25–7.31 (m, 2H), 7.34 (d, *J* = 8.0 Hz, 1H), 7.45 (d, *J* = 7.5 Hz, 1H), 7.65 (d, *J* = 8.0 Hz, 1H), 7.77 (td, *J* = 8.0, 1.5 Hz, 1H), 7.81 (td, *J* = 8.0, 1.5 Hz, 1H), 7.94 (dd, *J* = 8.0, 1.0 Hz, 1H), 8.05 (s, 1H), 8.49–8.55 (m, 2H), 9.17 (t, *J* = 6.0 Hz, 1H), 9.33 (t, *J* = 6.0 Hz, 1H); ^13^C NMR (125.7 MHz, DMSO-*d*_6_) 44.75, 45.00, 121.09, 121.22, 122.34, 122.38, 126.30, 128.54, 129.23, 130.28, 136.49, 136.92, 136.97, 139.20, 149.06, 149.07, 158.31, 158.54, 164.68, 166.26.

#### *N*-[4-chloro-3-(2-pyridinyl)phenyl]-4-[(methylsulfonyl)amino]-2-(trifluoromethyl)benzamide (MDB4)

MDB4 was synthesized as follows. To a mixture of 4-(methanesulfonylamino)-2-(trifluoromethyl)benzoic acid (312 mg, 1.1 mmol) and C_11_H_9_ClN_2_ (205 mg, 1 mmol), and DIPEA (129 mg, 1 mmol) in ACN (10 mL) was added HATU (570 mg, 1.5 mmol). The mixture was stirred at room temperature for 17 h and then evaporated to dryness. The crude product was purified by crystallization from EtOH/water (1:2) to give the desired product (384 mg, 82%) as a white solid. Mp 181–183 °C. ^1^H NMR (500 MHz, DMSO-*d*_6_) 3.13 (s, 3H), 7.42–7.51 (m, 1H), 7.51–7.59 (m, 3H), 7.67–7.75 (m, 3H), 7.93 (td, *J* = 8.0, 2.0 Hz, 1H), 8.00 (d, *J* = 2.5 Hz, 1H), 8.71 (d, *J* = 5.0 Hz, 1H), 10.40 (s, 1H), 10.75 (s, 1H).

#### 2-chloro-*N*^1^-[4-chloro-3-(2-pyridinyl) phenyl]-*N*^4^,*N*^4^-bis(2-pyridinylmethyl)-1,4-benzenedicarboxamide (MDB5)

MDB5 was synthesized in two steps. In the first step, to a mixture of C_9_H_7_ClO_4_ (540 mg, 2.5 mmol), C_12_H_13_N_3_ (740 mg, 3.7 mmol), and TEA (505 mg, 5 mmol) in ACN (25 mL) was added HATU (1.43 g, 3.75 mmol). The mixture was stirred at room temperature for 17 h and then evaporated to dryness. The residue was diluted with CH_2_Cl_2_ (50 mL) and extracted with brine (3 × 50 mL). The organic layer was dried over MgSO_4_, filtered, and concentrated. In the second step, a mixture of the crude amide and KOH (291 mg, 5.2 mmol) in CH_3_OH (25 mL) was heated at 50 °C for 17 h and cooled to room temperature. After removal of the solvent, the residue was purified by crystallization from ACN (50 mL) to give the desired potassium salt as a white solid. In step three, to a mixture of the potassium salt and 4-chloro-3-(pyridin-2-yl) benzenamine (197 mg, 0.96 mmol) in DMA (5 mL) HATU (547 mg, 1.4 mmol) was added. The mixture was stirred at room temperature for 17 h and then quenched with brine (50 mL). The resulting precipitate was collected by filtration and dried. The crude product was purified by chromatography (silica gel, EtOAc/ACN = 50:50) to afford the desired diamide (149 mg, 10%) as a white solid. Mp 89–91 °C. ^1^H NMR (500 MHz, DMSO-*d*_6_) 4.63 (s, 2H), 4.70 (s, 2H), 7.27–7.39 (m, 3H), 7.42–7.46 (m, 2H), 7.56 (d, *J* = 8.5 Hz, 1H), 7.62 (d, *J* = 7.0 Hz, 1H), 7.64–7.69 (m, 2H), 7.72–7.82 (m, 4H), 7.90–7.94 (m, 1H), 8.00 (d, *J* = 2.0 Hz, 1H), 8.53 (d, *J* = 3.5 Hz, 1H), 8.61 (d, *J* = 3.0 Hz, 1H), 8.70 (d, *J* = 3.5 Hz, 1H), 10.77 (s, 1H); ^13^C NMR (125.7 MHz, DMSO-*d*_6_) 50.17, 54.02, 121.03, 121.91, 122.38, 122.48, 122.56, 123.04, 123.15, 124.67, 125.72, 127.96, 129.25, 130.18, 130.48, 136.58, 137.10, 137.25, 137.36, 138.00, 139.25, 139.35, 149.21, 149.65, 149.72, 155.86, 156.02, 156.71, 164.69, 169.52.

#### 6-(4-acetyl-1-piperazinyl)-*N*-[4-chloro-3-(2-pyridinyl)phenyl]-2-methyl-3-pyridinecarboxamide (MDB6)

MDB6 was synthesized in two steps. In first step, to a mixture of C_7_H_6_ClNO_2_ (189 mg, 1.1 mmol), C_11_H_9_ClN_2_ (205 mg, 1 mmol), and DIPEA (129 mg, 1 mmol) in ACN (10 mL) was added HATU (570 mg, 1.5 mmol). The mixture was stirred at room temperature for 17 h and then quenched with water (50 mL). The resulting precipitate was collected by filtration and dried to give the desired amide (167 mg, 47%) as a white solid. In second step, a mixture of the intermediate (167 mg, 0.47 mmol) and C_6_H_12_N_2_O (179 mg, 1.4 mmol) in DMA (5 mL) was heated at 140 °C for 72 h. The reaction was cooled to room temperature and quenched with water (50 mL). The resulting precipitate was collected by filtration, rinsed with ACN (20 mL), and dried to give the desired product (169 mg, 80%) as a white solid. Mp 222–224 °C. ^1^H NMR (500 MHz, DMSO-*d*_6_) 2.05 (s, 3H), 2.48 (s, 3H), 3.51–3.65 (m, 8H), 6.75 (d, *J* = 9.0 Hz, 1H), 7.41–7.47 (m, 1H), 7.52 (d, *J* = 9.0 Hz, 1H), 7.67 (d, *J* = 8.0 Hz, 1H), 7.77 (d, *J* = 8.5 Hz, 1H), 7.81 (dd, *J* = 8.5, 2.0 Hz, 1H), 7.92 (t, *J* = 8.0 Hz, 1H), 8.02 (d, *J* = 2.0 Hz, 1H), 8.70 (d, *J* = 4.5 Hz, 1H), 10.34 (s, 1H).

### *In vitro* cell cytotoxicity assay

5 × 10^3^ MIA PaCa-2 and PANC-1 cells per well were seeded in 96-well plate and incubated in 5% CO_2_ at 37 °C in a 95% humidified atmosphere. After 24 h, the cells were treated with concentration gradients of GDC-0449 analogs (MDB1–6) (0–100 µM in DMSO) and cell viability was assessed by MTT (0.5 mg/mL) assay after 72 h^34^. 0.25% of DMSO was used as a control in all the experiments hereafter. Additionally, GDC-0449 and MDB5 loaded nanoparticles were also used for MTT assay.

To validate MDB5 to share same molecular pathway with GDC-0449, MIA PaCa-2 cells were seeded into 6-well plate at a density of 2 × 10^5^ cells/well in DMEM medium. At 70–80% cells confluency, ON-TARGETplus siRNA for Smo and ON-TARGETplus non-targeting pool as control siRNA (50 pmol/well) (Dharmacon, Inc.) was transfected in MIA PaCa-2 cells using Lipofectamine® RNAiMAX according to manufacturer’s instructions (Thermo Scientific, Rockford, IL). Post 24 h of transfection, cells were treated with 50 µM of drugs and cell viability was assessed by MTT. Labeled oligonucleotide (50 pmol/well) was compled with Lipofectamine® RNAiMAX and transfected according to manufacturer’s instructions and visualized under a fluorescent microscope.

### Molecular docking

The docking study of GDC-0449 analogs was carried out using Glide docking approach (Protein Preparation Wizard)^72^. The crystal structures of human Smo were retrieved through Protein Data Bank (PDB ID: 5L7I, 4QIM, 4QIN, 4O9R and 4N4W). These structures were superimposed using the Molecular Operating Environment program (MOE) (Chemical Computing Group Inc. Montreal, Canada) to identify the binding sites for subsequent docking studies. Proteins were prepared using the Protein Preparation Wizard at Maestro to flip the side-chain structures of residues Gln and Asn to maximize hydrogen-bond interactions. The grid file for 5L7I was generated using the Glide Grid Generation protocol with the bound ligand as the centroid. Ligand structures were constructed, minimized using the Maestro program, and subsequently docked to the model protein as defined in the grid file. During the docking process, the scaling factor for receptor van der Waals for the nonpolar-atoms was set to 0.8 to allow for a certain degree of receptor flexibility and the extra precision method was used. All other parameters were used as defaults. The binding affinity of the Smo/ligand complexes was expressed as docking scores, where the more negative docking score suggests more favorable ligand binding. The figures of protein/ligand interactions were generated with the Pymol program (Delano Scientific LLC, San Carlos, CA), and hydrogen atoms are hidden for clarity purposes.

Molecular docking in Smo mutants was carried by mutating the residues to respective mutant using MOE’s mutate function, following by energy minimization. After minimization, protein mutants were prepared using Schrodinger Maestro’s protein preparation module, and with side chain minimized using minimizers in Maestro. After that, grid files were generated using Glide dock protein, and then all ligands were docked to the grid files, which represent the binding pocket. After docking, ligands were evaluated based on their docking scores.

### Apoptosis and cell cycle analysis

3 × 10^5^ MIA PaCa-2 cells per well were grown in 6 wells plate for 24 h. After 24 h, the cells were treated with 50 µM of drugs. After 72 h post treatment, both live and dead cells were collected, washed twice with cold PBS and the pellets were suspended in 500 µL binding buffer containing 5 µL of annexin-V FITC and 1 µL of propidium iodide (100 µg/mL) (Alexa Fluor^®^ 488 annexin V Apoptosis Kit, Invitrogen). The mixture was incubated for 15 min in the dark at room temperature and analyzed by flow cytometry. For cell cycle analysis, after harvesting total cells, the pellets were suspended in 1 mL of cold 70% ethanol and incubated overnight at 4 °C for fixation. The next day, cells were washed twice with cold PBS and suspended in 500 µl FxCycle PI/RNase staining solution (Molecular Probes) and analyzed by FACS Calibur flow cytometer after incubated at 37 °C for 30 min in the dark.

### Colony formation and cell migration assay

The clonogenic assay was carried out by treating 1 × 10^3^ MIA PaCa-2 cells with 50 µM of drugs for 7 days. The cells forming the colonies were determined under a fluorescent microscope after staining with crystal violet. For migration study, 5 × 10^4^ MIA PaCa-2 cells were transferred into the upper chambers of the Transwell plates (8.0 μm transparent PET Membrane) in 2.5 ml DMEM supplemented with 1% FBS. The lower chamber was filled with 3 ml DMEM supplemented with 10% FBS. After 24 h of incubation at 37 °C under 5% CO_2_, cells were treated with drugs and further incubated for another 72 h at the same condition. The migrated cells were determined after staining with crystal violet and observed under a fluorescent microscope.

### Quantitative real-time RT-PCR and Western blot analysis

For quantitative real-time RT-PCR analysis, after 72 h of treatment with 50 µM of drugs, MIA PaCa-2 cells was lysed and the total RNA was extracted using RNeasy Mini Kit (QIAGEN, MD) followed by reverse transcription into cDNA^55^. Real-time RT-PCR was then performed to amplify cDNA using SYBR Green dye universal master mix on a Light Cycler 480 (Roche, Indianapolis) using the primers for Gli-1-F: 5′-CCAACTCCA CAGGCATACAGGAT-3′, R: 5′-CACAGATTCAGGCTCACGCTTC-3′; Gli-2-F: 5′-AAGTCACTCAAGGATTCCTGCTCA-3′, R: 5′-GTTTTCCAGGATGGAGCCACTT-3′; Shh-F: 5′-CCGGCTTCGACTGGGTGTACTA-3′, R: 5′-CGCCACCGAGTTCTCTGCTTT-3′; Smo-F: 5′-GCTACTTCCTCATCCGAGGAGTCA-3′, R: 5′-GGCGCAGCATGGTCTCGTT-3′; and GAPDH-F: 5′-ACCACAGTCCATGCCATCAC-3′, R: 5′-TCCACCACCCTGTTGCTGTA-3′ for forty cycles. For Western blot analysis, protein was isolated from MIA PaCa-2 and PANC-1 cells after treatment with 50 µM of drugs and concentration were determined using Micro BCA^TM^ Protein assay kit (Thermo Scientific, Rockford, IL). Equal amounts of protein were separated in 4–15% mini PROTEAN polyacrylamide gel followed by transferring to polyvinylidenefluoride (PVDF) (Life Technologies, Carlsbad, CA) membranes by iBlot gel transfer system. Membranes were blocked for 1 h at room temperature with blocking buffer containing 5% non-fat dry milk and 0.05% Tween-20 in 1X PBST. Membranes were incubated overnight with primary antibodies for Gli-1 (sc-20687), Shh (sc-9024), Bcl-2 (sc-492), Bax (sc-70406), ALDH1/2 (sc-166362), Oct-3/4 (sc-5279), GAPDH (sc-365062) (Santa Cruz Biotechnology, Inc.) and CD44 (3578) (Cell Signaling Technology, Inc.). Following washing with PBST, the membranes were incubated with their corresponding horseradish peroxidase-conjugated secondary antibodies (sc-2055, sc-2054 and sc-2056) (Santa Cruz Biotechnology, Inc.). Target proteins were detected by ImmunoCruz Western blotting luminol reagent kit (Santa Cruz Biotechnology, Inc.). To ascertain comparative expression and equal loading of the protein samples, the membrane stained earlier was stripped and re-probed with GAPDH and other target antibodies.

### 3D spheroid culture

For 3D spheroid generation, 3 × 10^3^ cells in 40 μl DMEM medium per well were seeded onto Perfecta3D® 96-Well Hanging Drop Plates (3D Biomatrix, Inc., Ann Arbor MI) in triplicate and incubated at 37 °C, 5% CO_2_ over the course of time. After the formation of 3D spheroid on day 3, the spheroids were treated with 50 µM of drugs on 4^th^ day and images were taken under a fluorescent microscope. Following treatment, images were taken at 3^rd^, 7^th^ and 9^th^ days to check the cytotoxic effect of drugs on 3D spheroid.

### Polymer synthesis and preparation nanoparticles

Methoxy-polyethylene-glycol-b-poly(carbonate-co-lactide) [mPEG-b-p(CB-b-LA)] copolymer was synthesized as described previously^70^. Nanoparticles carrying drugs were prepared using mPEG-b-p(CB-b-LA) by the emulsion/solvent evaporation technique^69^. The particle size distribution of nanoparticles was determined by dynamic light scattering using a Zeta Sizer^TM^ (Malvern 3800-ZLS, Boston, MA). Drug loading and encapsulation efficiency were determined by HPLC (Waters Corporation, Milford, MA). Briefly, GDC-0449, and MDB5 loaded nanoparticles with 0.5 mg theoretical drug loading were dissolved in 500 µL of DCM for drug extraction using a bath sonicator for 15 min at 37 °C. The solution containing DCM was collected, evaporated and the residues were dissolved in ACN. Drug content was determined by HPLC using a reverse phase C-18 Inertsil ODS column (150 mm × 4.6 mm, 5 μm) (GL Sciences Inc., Torrance, CA). Mobile phase composition was ACN and water (70:30) at a flow rate of 0.65 mL/min. Detection wavelength of 228 nm and 261.5 nm were used for GDC-0449 and MDB5, respectively.

### *In vivo* evaluation

All *in vivo* animal experiments were carried out as per the NIH animal use guidelines and protocol approved (#14-060-09-FC) by the Institutional Animal Care and Use Committee (IACUC) at the University of Nebraska Medical Center (UNMC), Omaha, NE. Tumor flank was established in 8–10-week-old male NOD.Cg-Prkdcscid Il2rgtm1wjl/SzJ (NSG) mice by subcutaneous injection of 3 × 10^6^ MIA PaCa-2 cells suspended in a total 50 µL of 1:1 PBS and Matrigel (BD Biosciences, San Diego, CA). When the tumor volume reached 100–120 mm^3^, animals were randomly divided into three groups (*n* = 5), i.e., control (PBS), nanoparticles containing GDC-0449 and nanoparticles containing MDB5. Formulations were administered intravenously thrice a week for 3 weeks at an equivalent drugs dose of 20 mg/kg. Tumor size was measured at regular intervals using a digital vernier caliper and body weight of the animals was recorded thrice a week. At the end of study, tumors were excised, weighed, and images were taken.

### Immunohistochemical staining and *in vivo* toxicity analysis

Mice tumor sections were stained with H&E, Ki-67, cleaved-caspase 3 and E-cadherin to determine toxicity, cell proliferation, apoptosis and EMT phenotype after drugs treatment. The toxic effects of MDB5 on major organs (kidney, spleen, liver, lung and heart) were determined by H&E staining. Positively stained (brown color) was selected for relative intensity.

For evaluation of short-term toxicity, when the volume of subcutaneous tumor reached 200 mm^3^, animals were randomly divided into two groups (*n* = 5), i.e., control (PBS) and MDB5 emulsion formulated by 65% dextrose solution and 35% of emulsifying agents (50% propylen glycol, 30% cremophore EL and 20% ethanol). Formulations were administered intraperitoneally thrice a week for 4 weeks at an equivalent drugs dose of 20 and 40 mg/kg. At the end of study, blood was collected in lithium heparin tubes (LH/1.3) via cardiac puncture and blood chemistry markers (hepatic) for each group were quantified following our earlier protocol^73, 74^.

### Statistical analysis

Student’s unpaired t-test was used to compare the mean values of individual groups. A p value < 0.05 was considered as statistically significant.

## Electronic supplementary material

Design, Synthesis and Biological Evaluation of novel Hedgehog Inhibitors for treating Pancreatic Cancer
