# Supplementary material for: Design, Synthesis and Biological Evaluation of novel Hedgehog Inhibitors for treating Pancreatic Cancer
Source: Sci Rep. 2017 May 10;7:1665. doi: 10.1038/s41598-017-01942-7 (PMC5431907; doi:10.1038/s41598-017-01942-7)
Supplement: Supplementary file 1 — Design, Synthesis and Biological Evaluation of novel Hedgehog Inhibitors for treating Pancreatic Cancer [file 41598_2017_1942_MOESM1_ESM.pdf]

Supplementary Information

Design, Synthesis and Biological Evaluation of novel Hedgehog Inhibitors for treating Pancreatic Cancer

Vinod Kumar[1,+], Amit Kumar Chaudhary[1,+], Yuxiang Dong[1], Haizhen A. Zhong[2], Goutam Mondal[1], Feng Lin[1], Virender Kumar[1] & Ram I. Mahato[1,*]

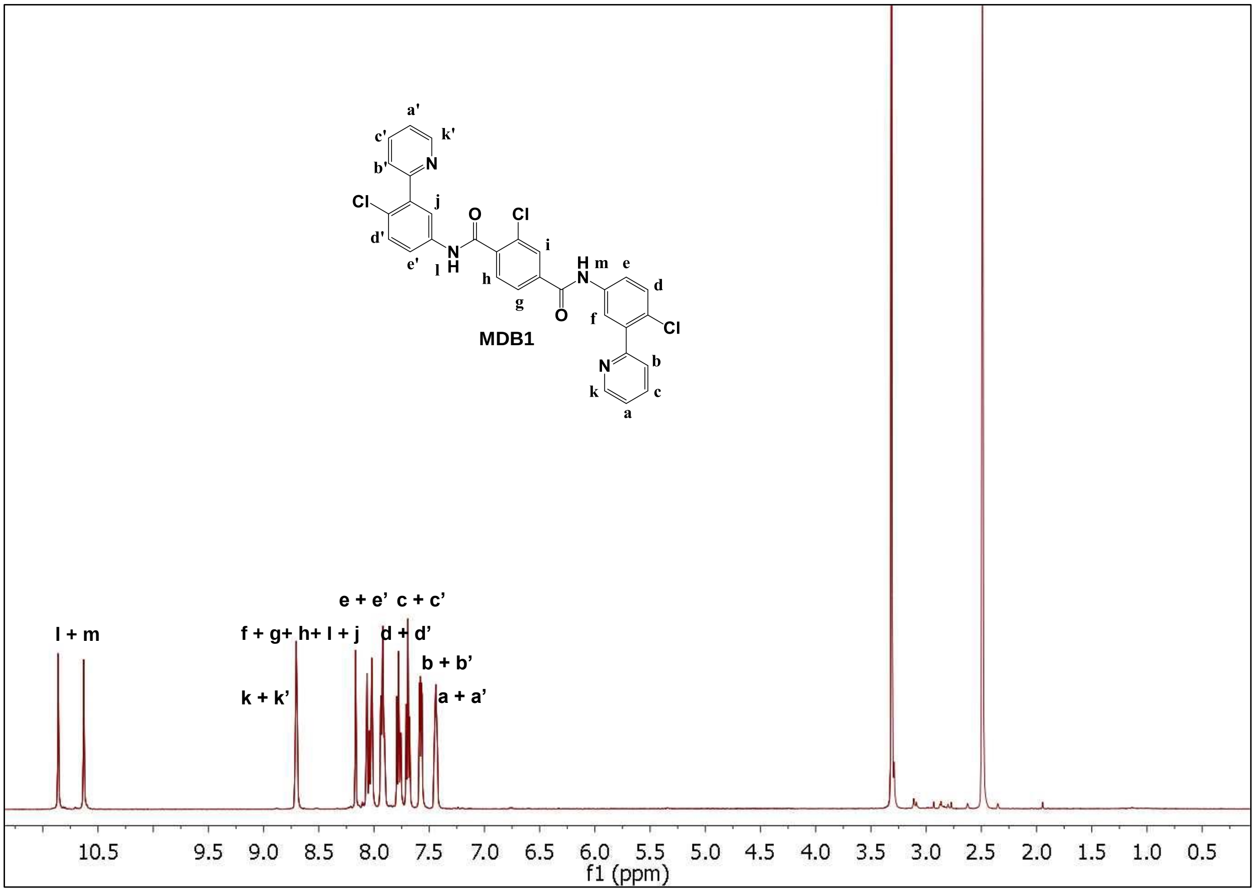

Fig. S1. ^{1}H-NMR of MDB1

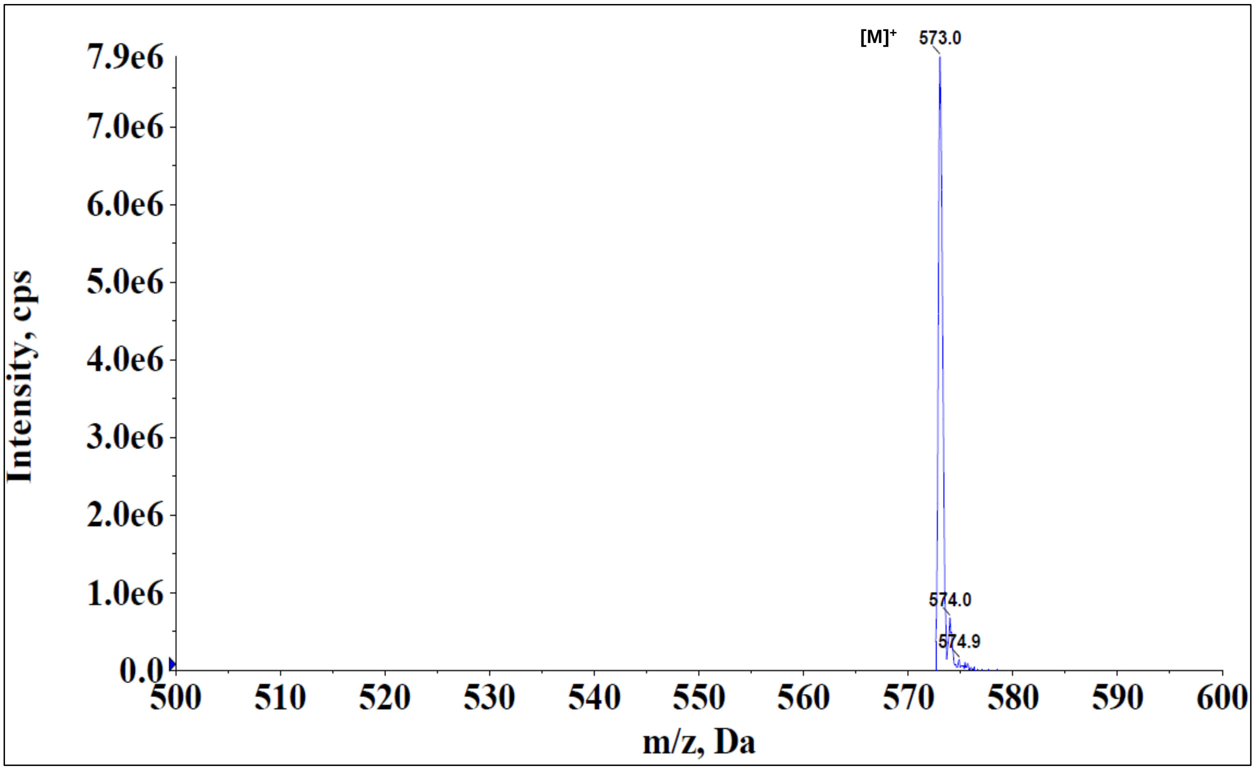

Fig. S2. QTRAP 4000 mass spectrometer of MDB1

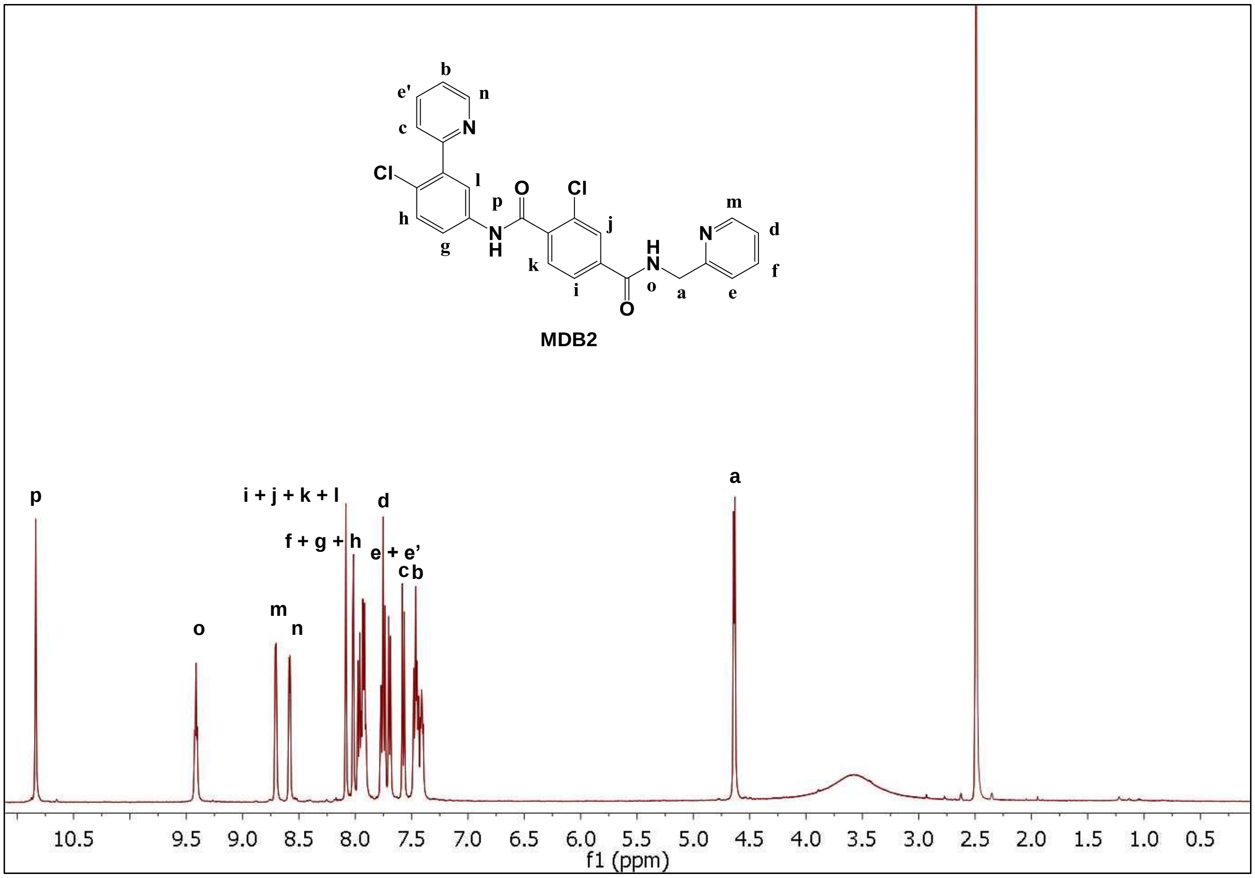

Fig. S3. ^{1}H-NMR of MDB2

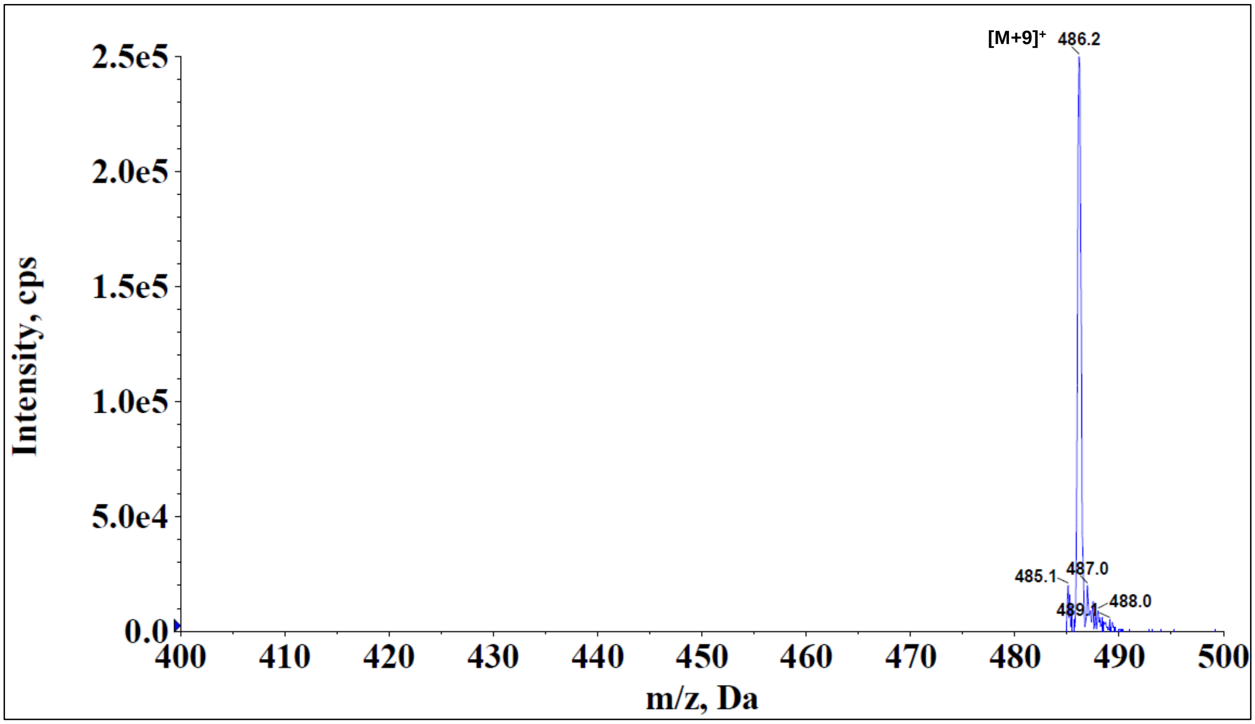

Fig. S4. QTRAP 4000 mass spectrometer of MDB2

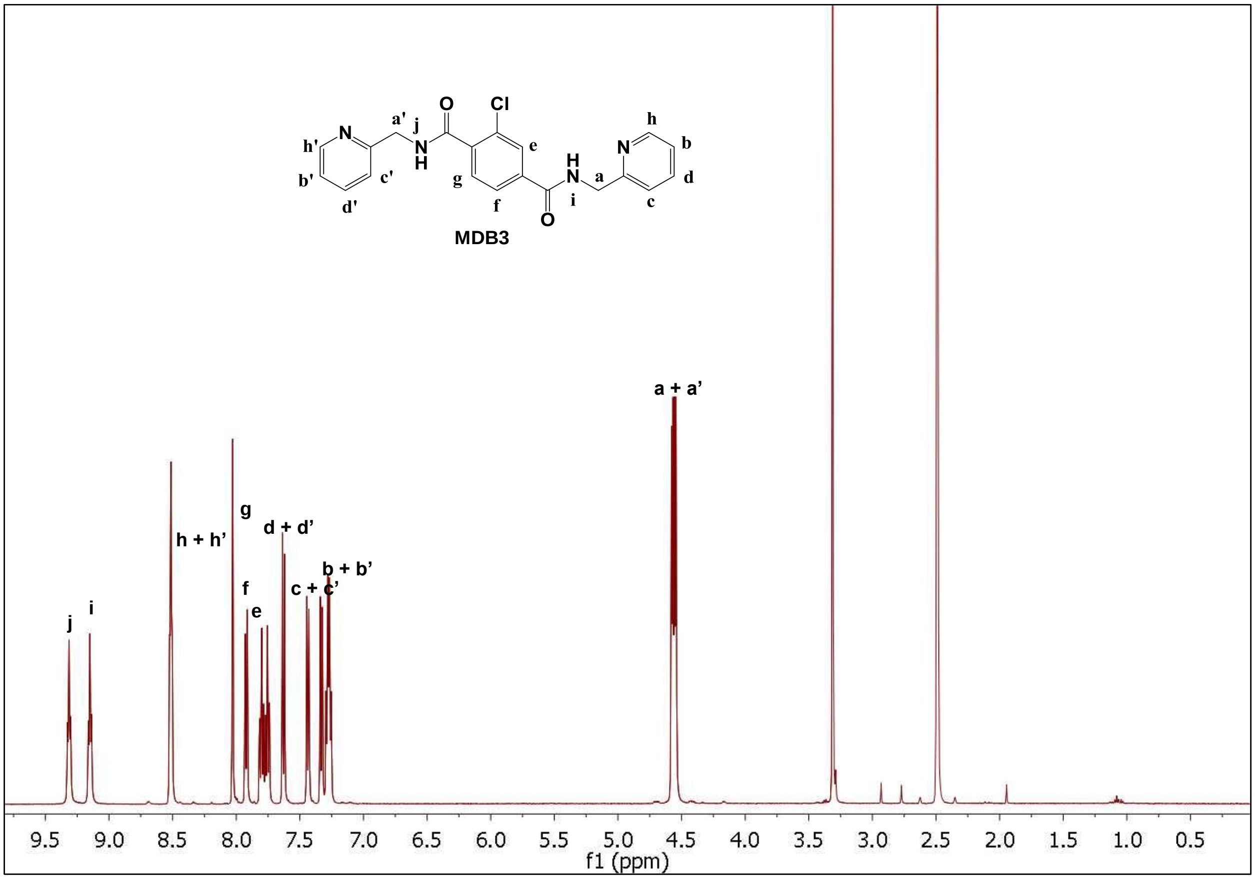

Fig. S5. ^{1}H-NMR of MDB3

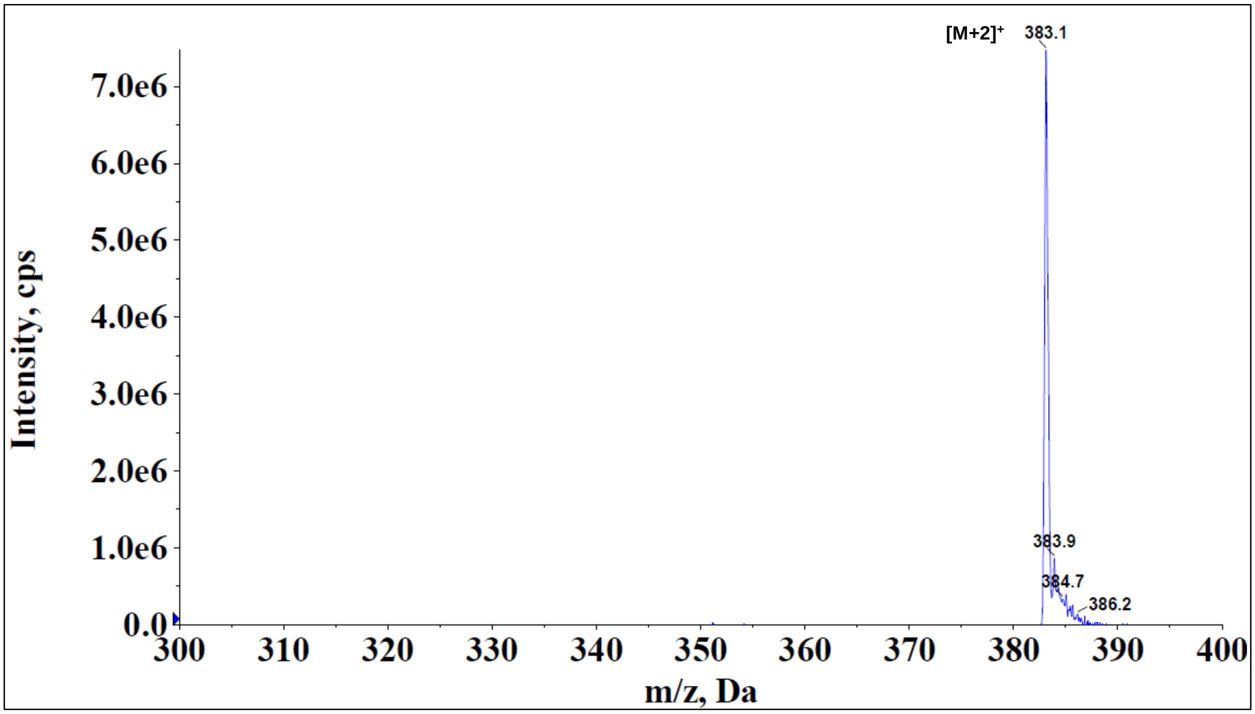

Fig. S6. QTRAP 4000 mass spectrometer of MDB3

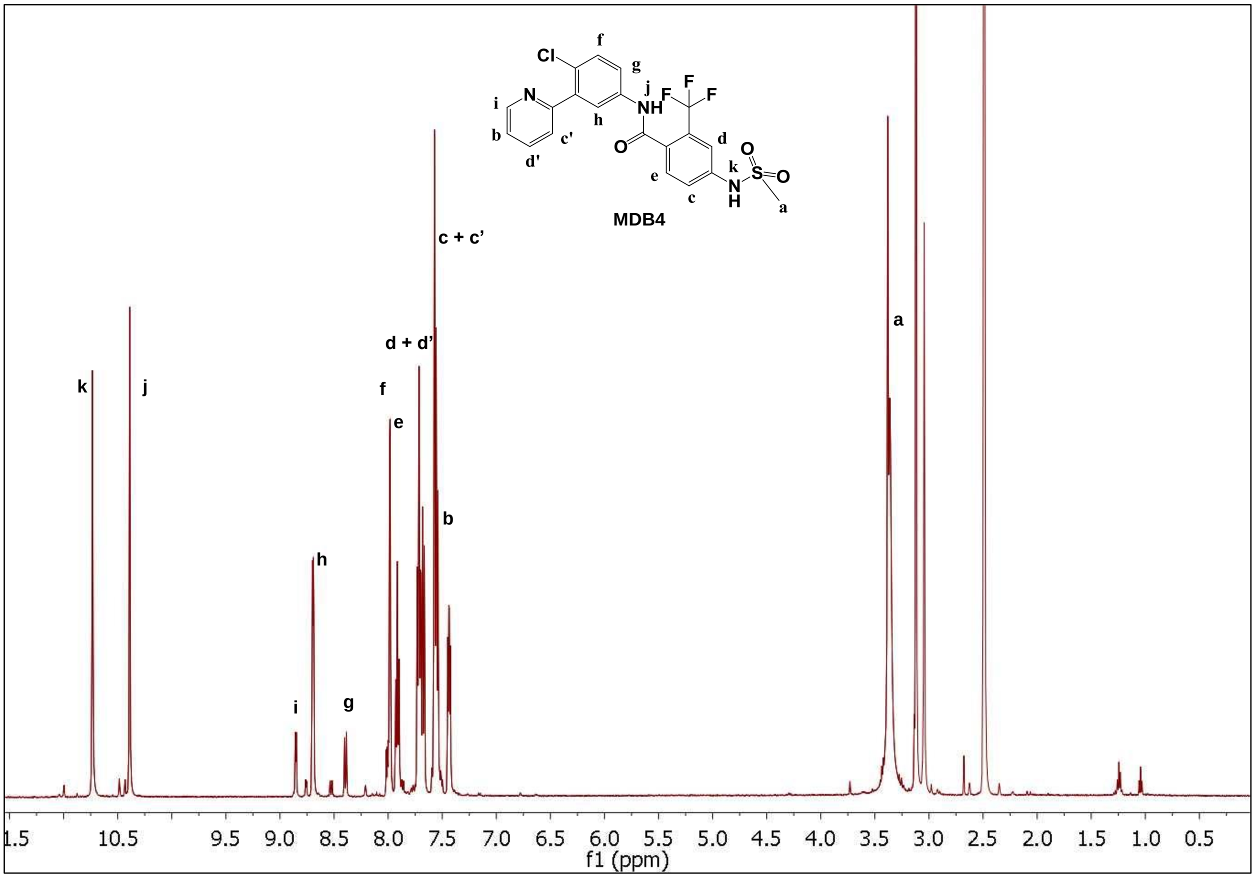

Fig. S7. ^{1}H-NMR of MDB4

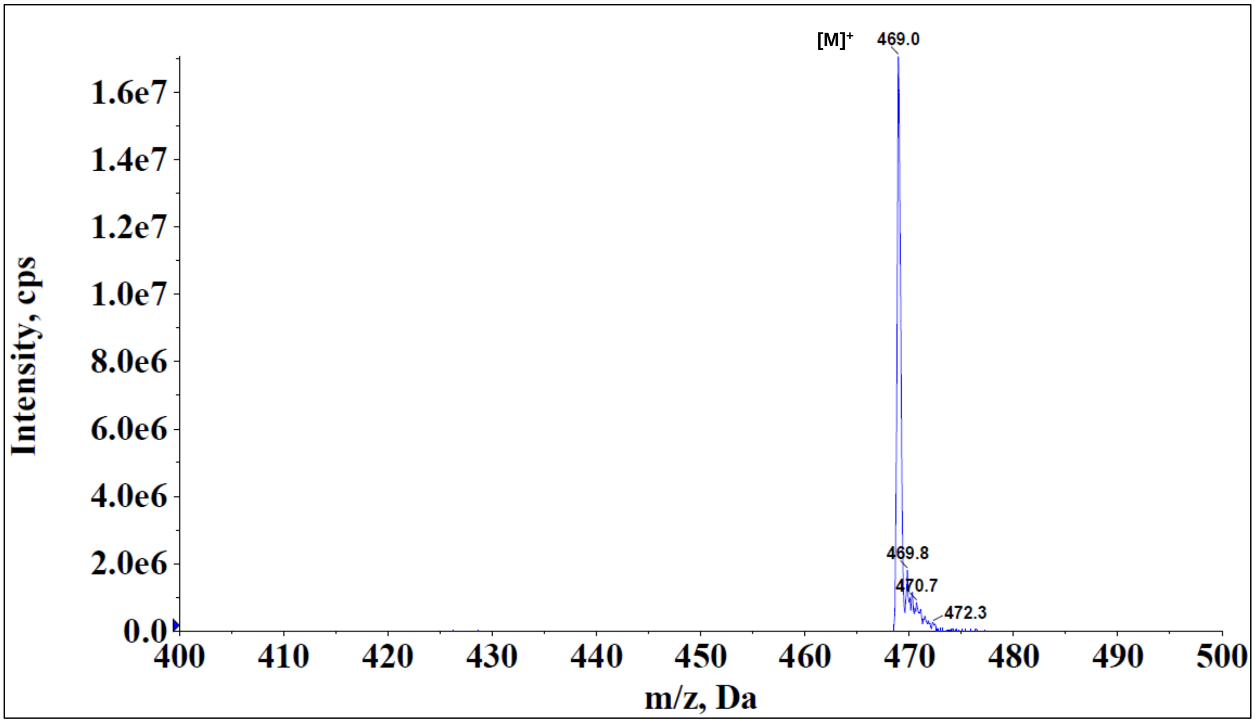

Fig. S8. QTRAP 4000 mass spectrometer of MDB4

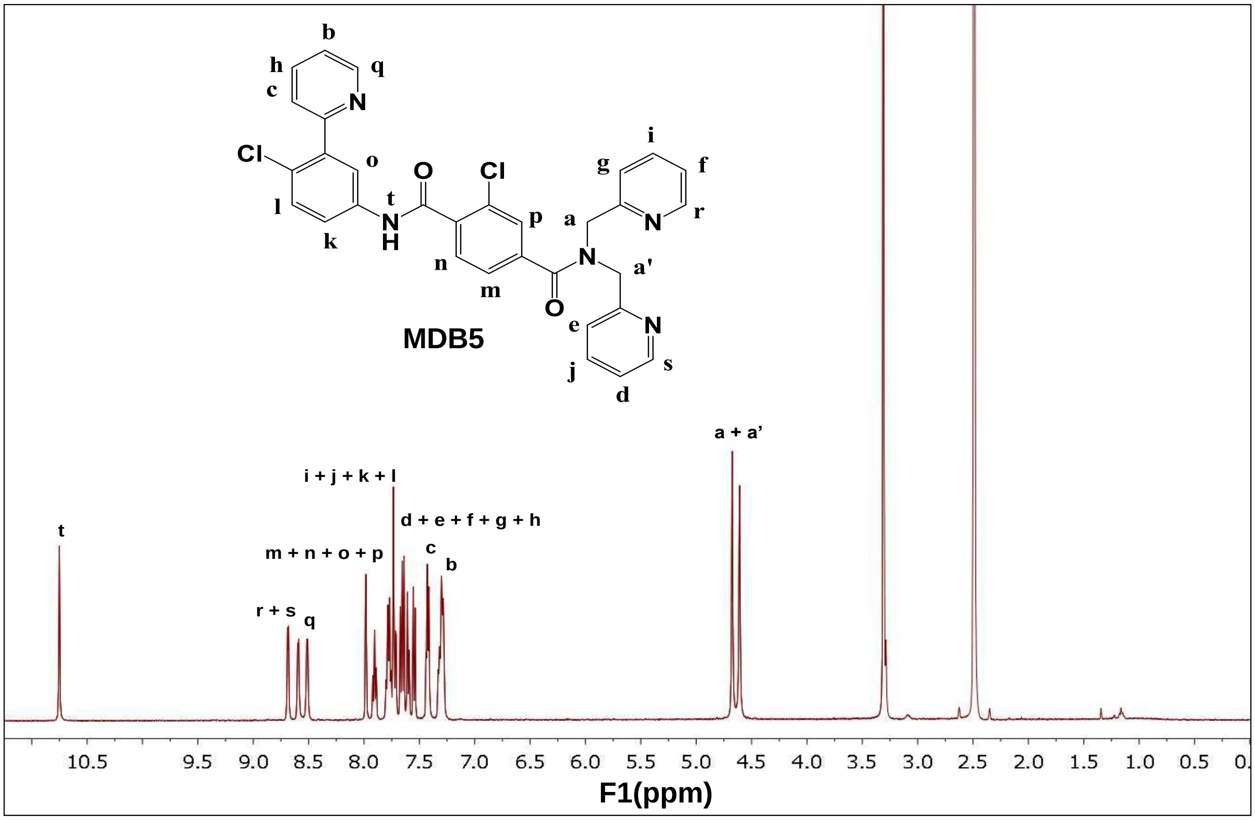

Fig. S9. ^{1}H-NMR of MDB5

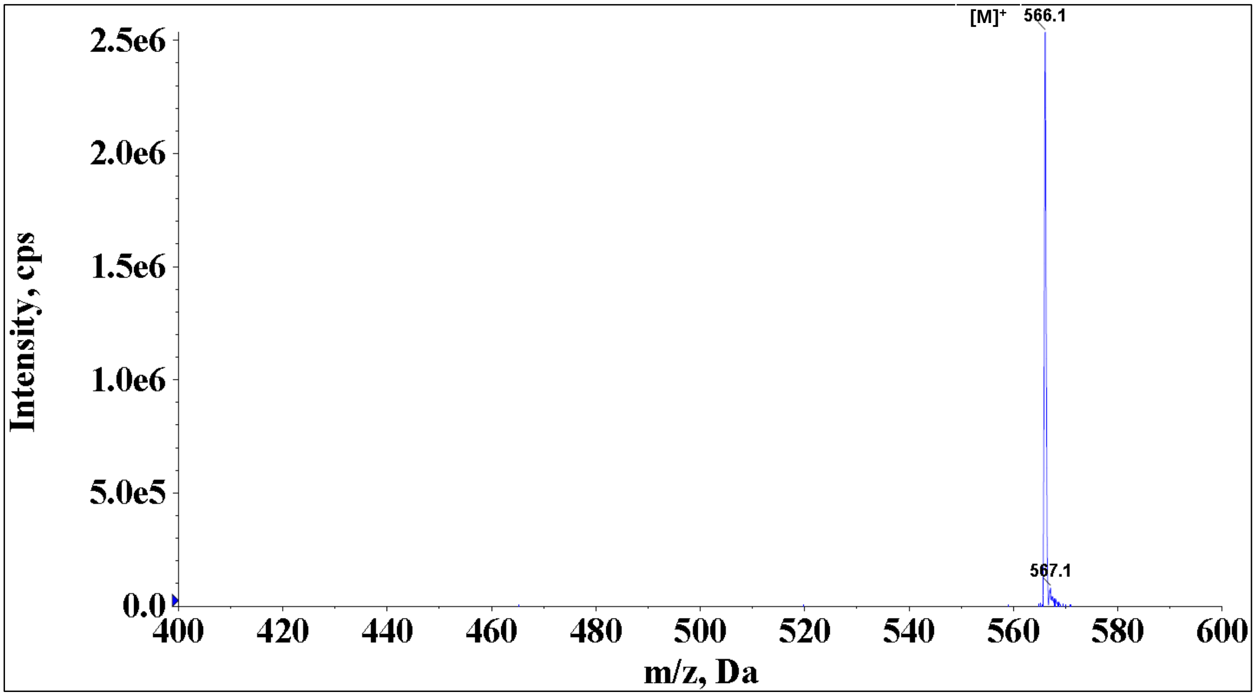

Fig. S10. QTRAP 4000 mass spectrometer of MDB5

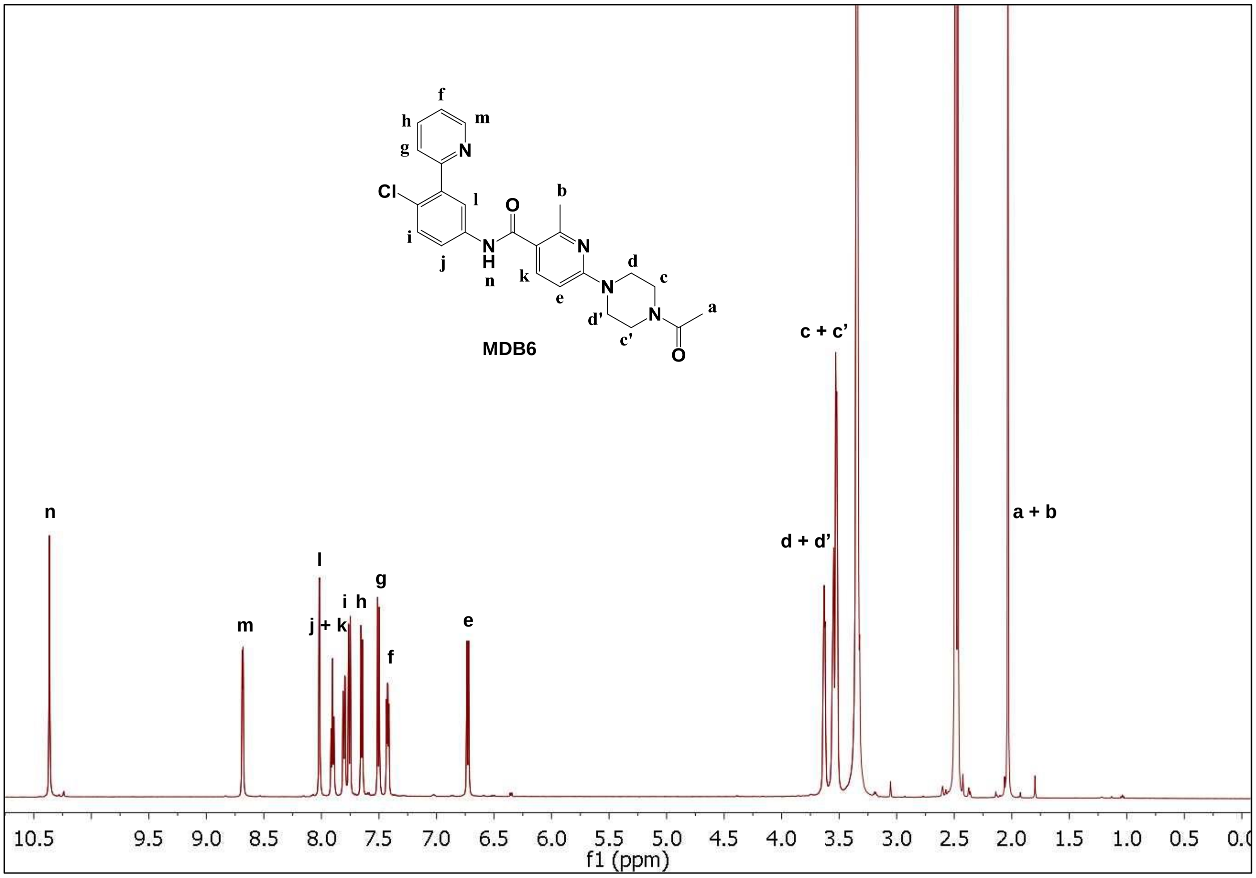

Fig. S11. ^{1}H-NMR of MDB6

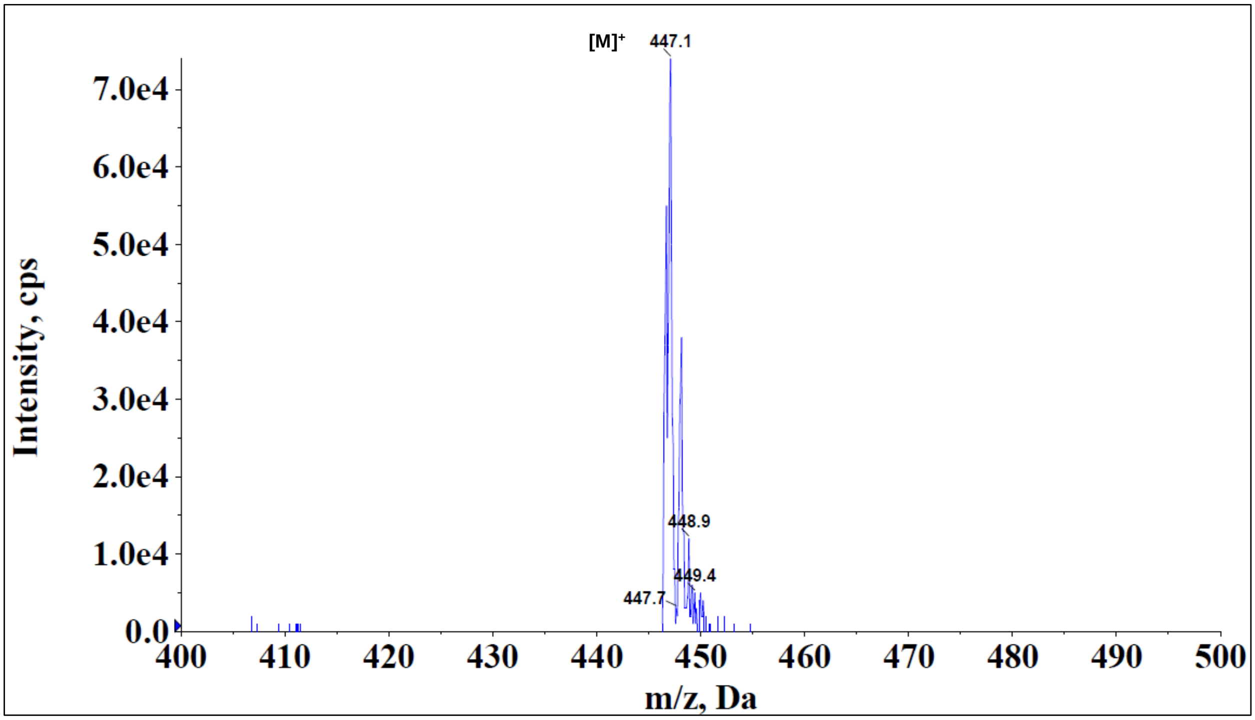

Fig. S12. QTRAP 4000 mass spectrometer of MDB6

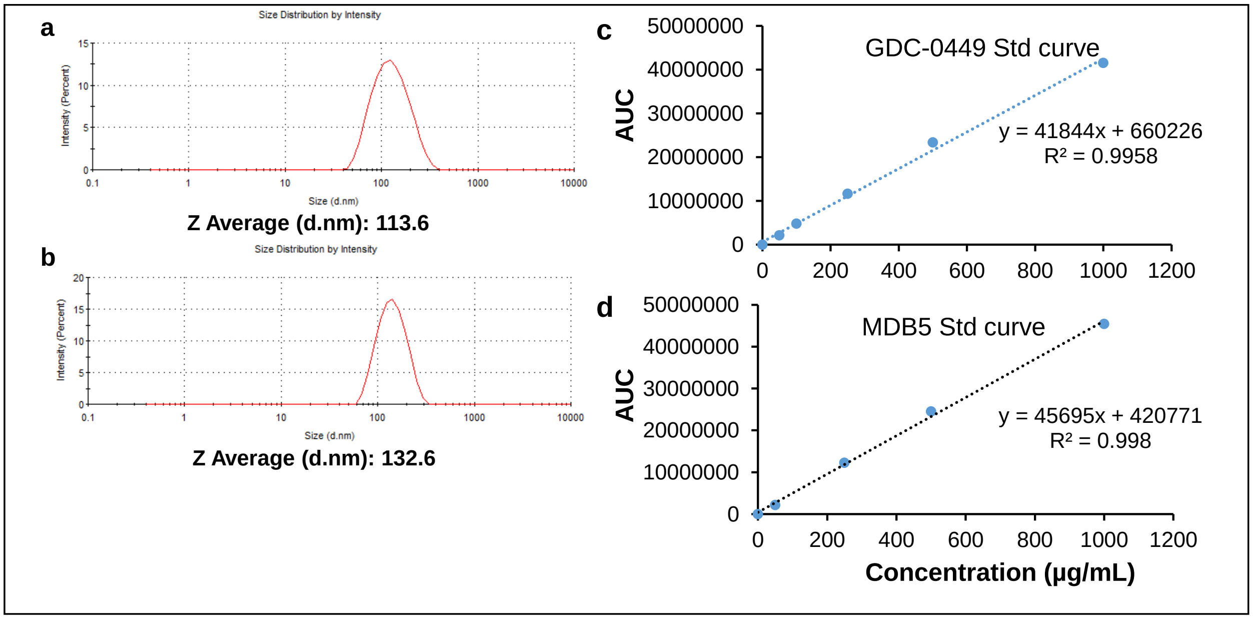

Fig. S13. Particle size distribution of **(a)** GDC-0449 and **(b)** MDB5 encapsulated nanoparticle using dynamic light scattering. Standard curve obtained from HPLC to calculate percentage of drug loading **(c)** for GDC-0449 and **(d)** for MDB5.

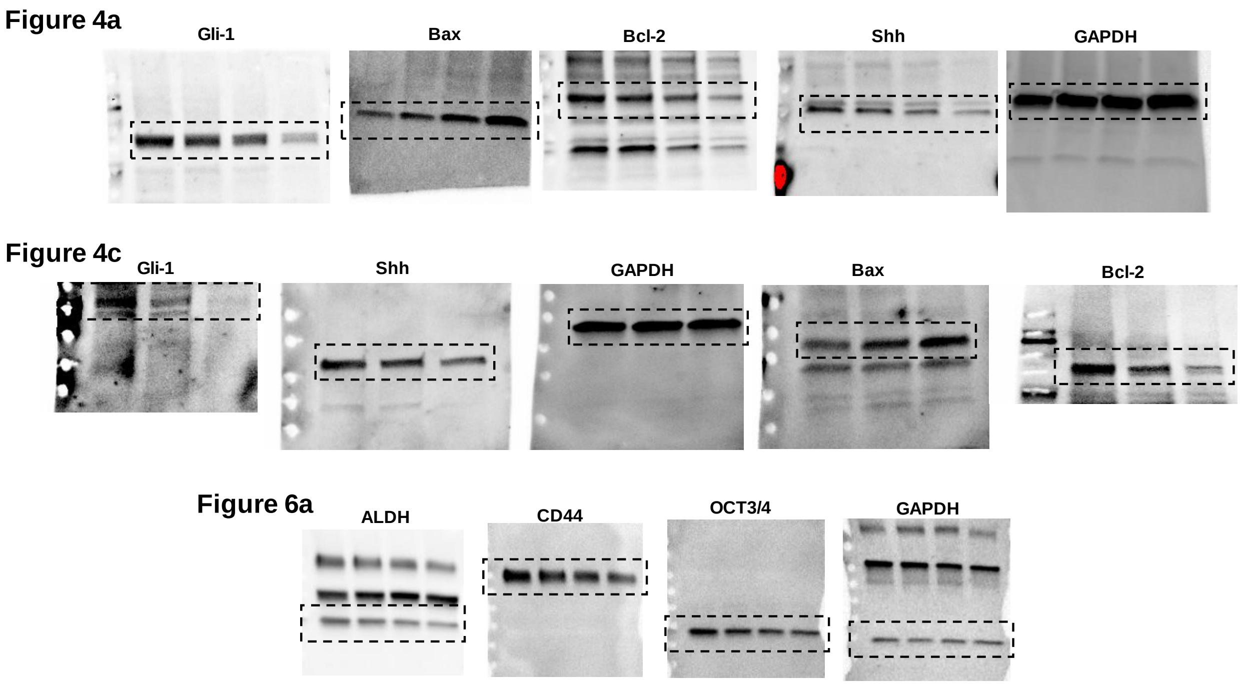

Fig. S14. Full-Length blots

Table S1. Blood chemistry.

Parameters	Control	20 mg/kg	40 mg/kg
Alanine Aminotransferase (ALT) U/L	26.4±3.91	32.0 ± 3.74	28.5 ± 6.35
Total Bilirubin (TBIL) mg/dL	0.28±0.04	0.22 ± 0.04	0.20
